# Bacteria contribute exopolysaccharides to an algal-bacterial joint extracellular matrix

**DOI:** 10.1038/s41522-024-00510-y

**Published:** 2024-04-01

**Authors:** Valeria Lipsman, Olesia Shlakhter, Jorge Rocha, Einat Segev

**Affiliations:** 1 Department of Plant and Environmental Sciences, Weizmann Institute of Science, Rehovot, 7610001 Israel; 2 Programa de Agricultura en Zonas Áridas, Centro de Investigaciones Biológicas del Noroeste, La Paz, Baja California Sur 23096 México

**Keywords:** Water microbiology, Biofilms

## Abstract

Marine ecosystems are influenced by phytoplankton aggregation, which affects processes like marine snow formation and harmful events such as marine mucilage outbreaks. Phytoplankton secrete exopolymers, creating an extracellular matrix (ECM) that promotes particle aggregation. This ECM attracts heterotrophic bacteria, providing a nutrient-rich and protective environment. In terrestrial environments, bacterial colonization near primary producers relies on attachment and the formation of multidimensional structures like biofilms. Bacteria were observed attaching and aggregating within algal-derived exopolymers, but it is unclear if bacteria produce an ECM that contributes to this colonization. This study, using *Emiliania huxleyi* algae and *Phaeobacter inhibens* bacteria in an environmentally relevant model system, reveals a shared algal-bacterial ECM scaffold that promotes algal-bacterial aggregation. Algal exudates play a pivotal role in promoting bacterial colonization, stimulating bacterial exopolysaccharide (EPS) production, and facilitating a joint ECM formation. A bacterial biosynthetic pathway responsible for producing a specific EPS contributing to bacterial ECM formation is identified. Genes from this pathway show increased expression in algal-rich environments. These findings highlight the underestimated role of bacteria in aggregate-mediated processes in marine environments, offering insights into algal-bacterial interactions and ECM formation, with implications for understanding and managing natural and perturbed aggregation events.

## Introduction

Microbial aggregation drives ecologically important processes in the marine environment^1^. The natural phenomenon of marine snow is caused by the aggregation of phytoplankton into small particles, driving the sinking of organic matter in the water column^2^. Marine snow plays a central role in surface-to-bottom fluxes, acting as a shuttle of nutrients from the photic zone to the bottom of the ocean^2^. In the Mediterranean seas, high nutrient influxes^3^, sea surface warming^4^ and water column stratification during summer^5^ are factors that can promote the merging of these algal aggregates into massive gelatinous layers, known as marine mucilage^5^. The mucilage poses several environmental threats, such as preventing oxygen diffusion to lower parts of the water column and carrying pathogenic bacteria^3,4^. However, the factors contributing to aggregation, including marine mucilage formation and the microorganisms involved, remain areas of limited understanding.

Aggregation creates a beneficial environment for the single cell by allowing cooperation between cells, and can lead to the development of multicellular life cycles^6^. Phytoplankton-derived polymers, such as exopolysaccharides (EPS), play a role in promoting aggregation. Among these polymers are transparent exopolymer particles (TEP), a type of acidic EPS enriched in sulfate half-ester groups, which confer high surface activity. As a result, TEP can contribute to the formation of a complex and interconnected extracellular matrix (ECM) and facilitate aggregation during phytoplankton blooms and in laboratory cultures^7^. Additionally, TEP can provide a surface for bacterial attachment and colonization, creating rich microenvironments by supplying bacteria with nutrients and a protective environment against predators^8^.

Bacteria were previously shown to promote the formation of algal-bacterial aggregates, by physical attachment to the algal cells and by stimulating algal TEP production^9^. Interestingly, increased aggregation of phytoplankton upon bacterial colonization is generally attributed to changes in phytoplankton TEP production. The challenges in tracing the origin of EPS within mixed algal-bacterial populations have often led to overlooking bacteria as potential contributors to microbial TEP formation. This is of particular interest since marine bacteria produce TEP by releasing capsular material and free EPS^7,10^. Yet, the active production of TEP by bacteria as part of the colonization process in the algal environment and its impact on algal-bacterial aggregation remains unclear.

In the terrestrial environment, there is comprehensive understanding of the importance of EPS produced by heterotrophic bacteria during their interaction with primary producers. Bacterial colonization of plant roots in the rhizosphere has been demonstrated to promote the formation of multicellular structures such as bacterial biofilms. Plant exudates secreted by the roots act as signals that promote chemotaxis by planktonic bacteria^11^. The early steps of colonization involve an initial, reversible attachment of bacteria to a surface, often stabilized by the secretion of specific bacterial proteins or EPS adhesins. The formation of a mature biofilm is characterized by a distinct spatial organization and the production of an adhesive ECM^12^. EPS is a key ECM component in most biofilms, and is often central in the formation of multidimensional structures that promote the colonization of plants.

In marine phytoplankton-bacteria interactions, namely between microalgae and abundant Roseobacter bacteria, much knowledge has been accumulated about the algal-bacterial metabolic exchange^13^. However, our understanding of algal-bacterial aggregation is partial at best. A handful of studies addressed the process of bacterial colonization and direct attachment to algal cells^14,15^. In these previous studies, the term biofilm is largely used to describe bacterial attachment and not an ECM-held multidimensional structure. Many uncertainties remain regarding bacterial colonization and algal-bacterial aggregation, particularly when drawing insights from terrestrial systems. Despite exhibiting resemblances to other models of primary producer-bacterial associations, the ability of marine bacteria to form multidimensional ECM-based structures in response to algal signals is yet to be established. Additionally, the composition and source of the ECM responsible for agglomerating algal-bacterial aggregates remain unknown.

Here, we use an algal-bacterial pair that naturally co-occurs in the marine environment^16^ to study their aggregation and ECM. Our model system includes the algal strain *Emiliania huxleyi* (also termed *Gephyrocapsa huxleyi*^17^) and the bacterial species *Phaeobacter inhibens*. This algal-bacterial model system was previously studied by us^18^ and by others^19,20^, revealing many routes of interactions and their environmental importance. Bacteria of the *P. inhibens* species attach through their pole to both abiotic and biotic surfaces^14,21^, including direct attachment to *E. huxleyi* cells^18^. Although attachment is the first step towards forming multidimensional ECM-held structures^12^, whether *P. inhibens* can produce ECM-held biofilms subsequent to attachment is unknown. We aimed to understand the process of algal-bacterial aggregation by addressing the key steps of this process. We investigated the algal impact on the bacterial transition from free-living cells to colonizers of the algal environment. Additionally, we examined whether this colonization process involves the production of a bacterial ECM. Furthermore, we analyzed the bacterial role in forming a joint algal-bacterial ECM and its impact on aggregation. Elucidating these central aspects of algal-bacterial aggregation can provide insight into natural phenomena such as marine snow, and enhance our understanding about instances of perturbed conditions that result in harmful aggregation, like marine mucilage.

## Results

### Algal exudates promote bacterial surface attachment

Phytoplankton exudates create concentrated hotspots in seawater^22^, attracting motile bacteria through chemotaxis. High concentrations of these chemoattractant in proximity to the exuding cell can promote aggregation around the algal cell^23,24^. It therefore appears plausible that the localized high concentrations of exudates in the phycosphere could induce a bacterial shift from a motile to a sessile lifestyle, characterized by surface attachment. *P. inhibens* was previously shown to be attracted^19^ and to aggregate^18,19^ in response to molecules exuded by *E. huxleyi*^19^. To mimic an environment of an algal population that is actively growing and metabolically attractive for bacterial colonization, an exudate-containing filtrate (termed spent medium) was collected from exponentially growing *E. huxleyi* monocultures. To study the effect of algal exudates on the gradual process of the bacterial transition from motility to surface attachment, we introduced the algal spent medium into *P. inhibens* bacterial cultures at early growth stages. Spent medium was introduced at different concentrations to assess a concentration-dependent effect (0–100% v/v). Prior to any treatment, the bacterial media already contained carbon, nitrogen, phosphorus, and sulfur needed for bacterial growth (see CNPS in materials and methods). Henceforth, we will refer to this bacterial medium as ASW medium, and the spent medium treatment will be incorporated into it. Bacterial attachment was assessed during different growth phases (mid exponential, early stationary and stationary) using an attachment assay that evaluates attachment to an abiotic 96-well plate^25^.

**Fig. 1:**
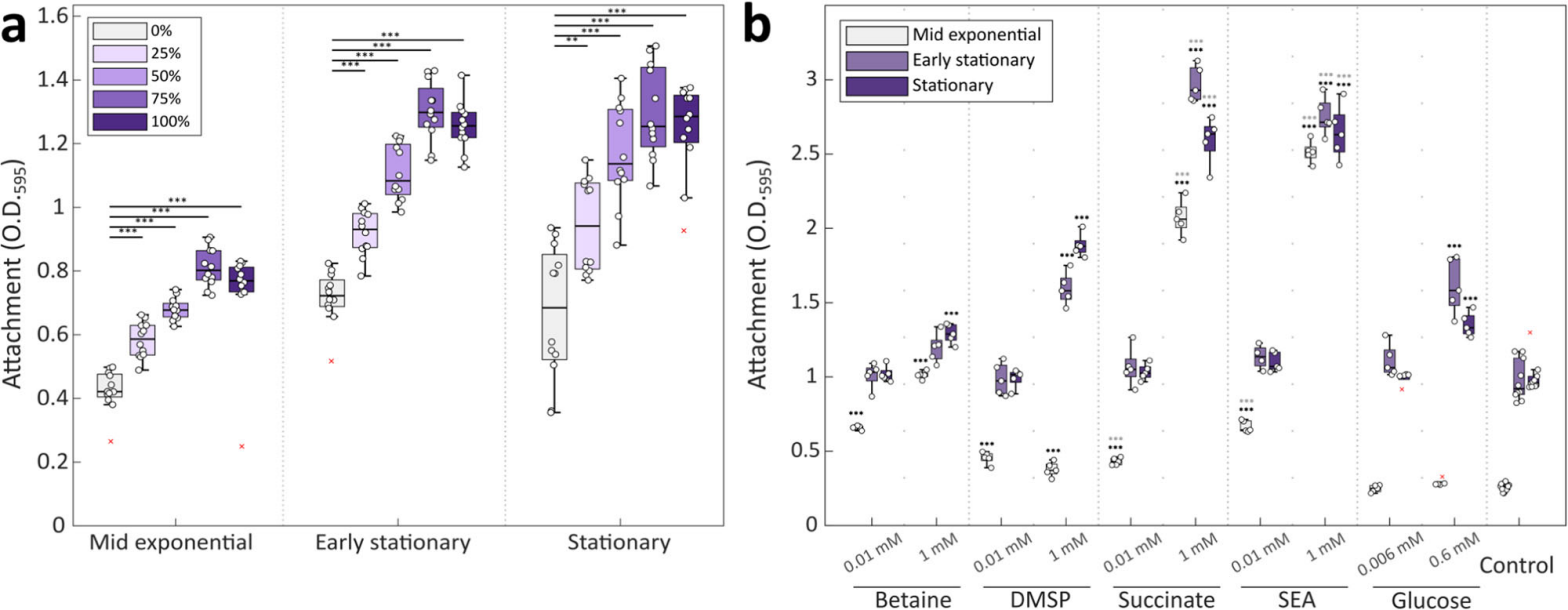
Algal exudates promote bacterial surface attachment. *P. inhibens* bacteria were grown with different supplements to evaluate their effect on attachment to an abiotic surface. Following a washing step, attachment was assessed by measuring the absorbance at 595 nm of the crystal violet dye extracted from bacterial cells that were strongly attached to 96-well plates^25^ (see Materials and Methods). **a** Bacteria supplemented with varying concentrations of algal spent medium (ranging from 0% to 100% v/v of spent/ASW medium), retrieved by filtering exponentially growing *E. huxleyi* monocultures. Each box consists of *n* = 12 wells. **b** Bacteria supplemented with varying concentrations of betaine, DMSP, succinate, or a mix containing either 0.01 mM or 1 mM of each compound (SEA). A control group without any added compounds was included. A control of additional glucose was introduced at 0.006 mM or 0.6 mM, providing additional carbon equivalent to the succinate treatment. Each box consists of *n* = 5 wells. Box-plot elements are: center line—median; box limits—upper and lower quartiles; whiskers—min and max values, red x—outliers. Statistical significance was determined using ANOVA, followed by the post-hoc Bonferroni test. Black asterisks indicate treatments where attachment significantly exceeded that of the control at the same bacterial growth phase. Gray asterisks (in panel **b**) denote treatments with succinate or SEA mix that exhibited significantly higher attachment compared to glucose treatments at the corresponding bacterial growth phase. Two or three asterisks represent p-values lower than 0.01 or 0.001, respectively.

Bacteria supplemented with algal exudates showed increased attachment compared to control cultures (Fig. 1a). Algal spent medium significantly increased bacterial attachment at all bacterial growth phases and across a range of exudate concentrations. Furthermore, during mid exponential and early stationary growth, bacterial attachment linearly correlated with the increase in algal exudate concentrations between 0–75%. These findings suggest that bacterial attachment in response to algal cues is concentration-dependent, similar to chemoattraction^26^.

Algal spent medium contains metabolites that can facilitate bacterial growth^27^. In our experiment, algal exudates were introduced into a medium that contains everything needed for bacterial growth. Nevertheless, we wished to test whether improved bacterial attachment is the result of increased growth. Therefore, we monitored bacterial growth until stationary phase in cultures supplemented with algal exudates versus control cultures. Our data shows that bacteria supplemented with spent medium exhibited earlier transition to logarithmic growth as previously reported^28^ (Supplementary Fig. 1), however these cultures did not exhibit higher yields in comparison to control cultures. Rather, slightly lower yields were observed upon treatment with algal exudates. Thus, it appears that bacterial enhanced attachment in response to algal exudates is dose-dependent, and the improved attachment does not stem from increased bacterial growth.

### A synthetic mixture of algal exudates promotes bacterial attachment in a concentration-dependent manner

Spent medium is a complex mixture of secreted algal compounds. Once collected, the spent medium contains the bulk average of algal exudates and fails to recapitulate important nuances such as elevated exudate concentrations that bacteria experience in proximity to the algal cell. To study the effect of high levels of algal exudates on bacterial attachment, we supplemented bacteria with selected algal exudates to allow precise manipulation of concentrations. The algal exudates dimethylsulfoniopropionate (DMSP), betaine and succinate, were previously monitored in laboratory algal cultures and in the environment^24,29,30^. Importantly, these compounds were previously shown to influence the chemotactic behavior in various marine bacteria^24,26^. Close to phytoplankton cells, microzones of high exudate concentrations are formed. However, concentrations rapidly decrease by orders of magnitude as the distance from the cell increases towards the bulk seawater^26^. To represent the chemical environment that bacteria may experience in different distances from the algal cell, bacterial cultures were supplemented with individual compounds in concentrations that represent proximity (1 mM) or distance from the algae (0.01 mM), and attachment to 96-well plates was assessed.

Low concentrations of the individual compounds increased bacterial attachment when the treated bacterial cultures were at mid-exponential phase (Fig. 1b). High concentrations of these compounds further increased bacterial attachment to levels greater than those observed upon treatment with algal spent medium (Fig. 1a). As these compounds can serve as an additional carbon source for bacteria^31–33^, we tested whether increased attachment was achieved due to additional carbon availability. Therefore, bacterial cultures were supplemented with additional glucose, in concentrations that reflect the carbon content of the succinate treatment, which showed the most pronounced improvement of attachment (Fig. 1b, 0.006 mM and 0.6 mM glucose, for low and high concentrations, respectively). Importantly, our prior research has demonstrated that glucose serves as an utilizable carbon source capable of supporting *P. inhibens* growth as a sole carbon source^18^. Results of these experiments demonstrate that the added glucose did not improve attachment beyond the impact observed by the added succinate. Thus, under our experimental conditions, succinate is likely to act as an attachment-promoting signal rather than a carbon source. Moreover, upon treatment with succinate, bacteria did not reach higher yields compared to control cultures (Supplementary Fig. 2), further demonstrating that the improved attachment is not due to increased growth.

In the environment, bacteria encounter a mixture of molecules exuded by algae. Therefore, we assembled a synthetic mixture with the representative algal exudates (SEA- Synthetic Exudates of Algae) that contained either 0.01 mM or 1 mM of each individual compound. Bacteria exposed to high concentrations of the SEA mix showed significantly higher attachment at mid exponential growth, beyond the levels observed for individual compounds. Interestingly, treatment with individual compounds and with the SEA mix resulted in shorter lag phases similar to the treatment with algal exudates, though bacterial yields exhibited slight variations. Thus, bacterial attachment to an abiotic surface is promoted by high concentrations of synthetic algal exudates, akin to the chemical environment in the proximity of the algal cell.

### The synthetic algal exudate array increases the production of bacterial EPS

Bacterial EPSs often play a key role in processes of attachment and aggregation^34^. To determine whether bacterial EPSs are involved in agglomerating SEA-treated bacteria, we stained bacterial samples grown in liquid medium with Alcian Blue, which targets acidic polysaccharides^7^. Our results show that SEA-treated bacteria produce extracellular sheet-like structures that are stained by the dye, while these structures are not observed in the control cultures (Supplementary Fig. 3a). Moreover, quantification of the extracellular carbohydrates extracted from SEA-treated bacteria showed significantly higher amounts of EPS compared to cultures of untreated bacteria (Supplementary Fig. 3b). Therefore, it is likely that bacterial EPS production is induced by the SEA treatment, suggesting a role in facilitating attachment and aggregation.

### The synthetic algal exudate mixture promotes the expression of bacterial genes related to EPS production

SEA mix treatment promoted the bacterial transition from a motile lifestyle to attachment and stimulated EPS production. To gain insights into these processes, the expression of the genetic modules that underlie these bacterial transitions was examined. To identify indicative genes that can serve as markers for each process, a comprehensive literature search was conducted (Supplementary Table 1). The genes *motA* and *motB* were selected for motility markers, having an established role in bacterial motility across various bacteria^35^. The gene *dltA1* was selected as a marker for initial surface attachment, encoding for a protein shown to be involved in surface attachment of bacteria such as *Staphylococcus aureus*, through the biosynthesis of d-alanyl-lipoteichoic acid (LTA)^36^. Furthermore, DltA deficiency was shown to reduce biofilm formation in *S. aureus* by reducing primary adhesion capabilities^36^. Although LTA is specific to Gram-positive bacteria, a few Gram-negative bacteria like *P. inhibens* carry this gene and its possible function in marine bacteria was previously discussed^37^. *P. inhibens* also carries several genes previously annotated as belonging to the biosynthetic pathway of succinoglycan, an EPS known to be produced by *Rhizobium* and *Agrobacterium* species^38^. As markers for EPS production, we chose the genes annotated as *exoB*, and two copies of *exoY*, encoded on the chromosome and the 262 kb plasmid of *P. inhibens*. Both genes encode for proteins that are responsible for the priming of EPS biosynthesis: *exoB* encodes for a protein producing the activated nucleotide-sugar UDP-galactose, which is used by the protein encoded by *exoY* to initiate the primary sugar chain biosynthesis^38^.

**Fig. 2:**
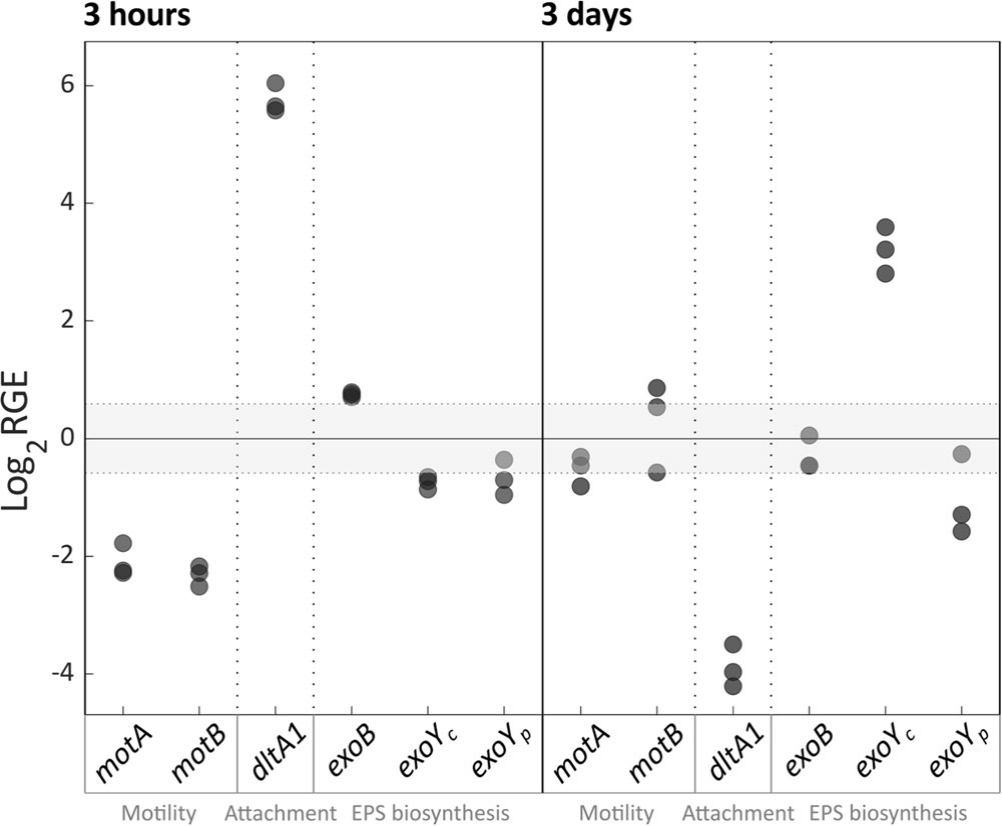
The SEA mix increases expression of bacterial genes potentially involved in attachment and EPS production. The relative gene expression (RGE) of genes involved in motility, attachment, and production of EPS was measured 3 hours (mid exponential, left) or 3 days (stationary, right) after addition of the SEA mix. Gene expression was normalized to two housekeeping genes (*gyrA* and *recA*) and to untreated samples collected at the same time. RNA was collected from three biological replicates for both SEA-treated and untreated bacteria grown in liquid medium. Upregulation or downregulation was determined according to a threshold of ±1.5 RGE. The chromosomal and plasmid copies of the *exoY* gene are denoted as *exoY*_*C*_ and *exoY*_*P*_, respectively.

Using qRT-PCR, we followed the expression of the marker genes over time in bacteria growing in liquid medium and subjected to SEA mix treatment. Soon after supplementing the SEA mix (3 hours, during mid exponential growth), the genes *motA* and *motB* were downregulated, while the genes *dltA1* and *exoB* were upregulated (Fig. 2). At stationary phase (3 days), the chromosomal *exoY* was upregulated, while the *dltA1* gene was downregulated. Interestingly, only the chromosomal *exoY* was upregulated, and not the copy found on the plasmid, suggesting different regulation of these two gene copies. Thus, the SEA mix appears to induce a behavioral switch in bacteria. The synthetic exudates initially trigger surface attachment while downregulating motility, and later promote the production of a specific EPS component.

### Bacterial gene modules involved in EPS production are expressed in algal-bacterial co-cultures

We next followed the processes involved in the bacterial transition from motility to attachment and EPS production in the context of algal-bacterial interactions. First, we curated the genetic modules that are potentially involved in the EPS biosynthetic pathway in *P. inhibens*. Functional annotation, using EggNOG-mapper v2^39^, revealed gene clusters around the two *exoY* genes (Supplementary Fig. 4a), resembling other characterized wzx/wzy-dependent EPS modules^40^ (Supplementary Table 2). These EPS modules harbor genes responsible for priming glycotransferases (e.g., *exoY*), facilitating the transfer of activated nucleotide-sugars to a lipid carrier and initiating EPS biosynthesis on the cytoplasmic side of the inner membrane^40^. Subsequently, various glycotransferases (GT) transfer diverse sugar bases, constructing the primary sugar chain^40^. The *P. inhibens* plasmid cluster contains several GTs orthologous to succinoglycan GTs, whereas the chromosomal cluster harbors a variety of GTs distinct from those in characterized EPS. This suggests that the two *P. inhibens* clusters encode different EPSs with varying chemical structures. The complete sugar chain unit is translocated to the periplasm by a flippase (wzx) and polymerized by the repeating unit polymerase (wzy), along with a polysaccharide co-polymerase (PCP, wzz), which is considered to control chain length^40^. Both *P. inhibens* clusters carry genes related to the machinery required for chain translocation and polymerization, suggesting that each EPS is synthesized via a distinct module-specific pathway. The final EPS is secreted by an outer membrane polysaccharide export protein (OPX)^40^. The *P. inhibens* plasmid cluster contains a gene encoding an OPX, however it is absent in the chromosomal cluster. While OPX is generally found within the EPS cluster, instances where an OPX homolog is found in a different locus were described^41^. *P. inhibens* indeed carries an OPX homolog in a different chromosomal location. However, whether the chromosomally encoded EPS is secreted via this gene product remains to be determined. Many characterized EPS modules also carry genes related to sugar base modification, such as acyl group addition^40^. While genes linked to sugar base modification were not detected around the two *P. inhibens* clusters, the variability in the accessory moieties and the corresponding gene variability seen in EPSs could explain the absence of clear orthologs in *P. inhibens* EPS modules^40,42^.

Following the curation of EPS-related genes, we sought to explore their expression in algal-bacterial co-cultures. Previously, our lab has generated transcriptomic data of *P. inhibens* bacteria in co-culture with *E. huxleyi* algae^43^. These data revealed that bacterial genes encoding for functions related to EPS biosynthesis are expressed in co-cultures with algae, as indicated by detectable levels of transcripts (Supplementary Fig. 4b). Specifically, during exponential growth and stationary phase, upregulation of the expression of the *exoY*-like gene from the chromosomal module was evident in bacteria grown in co-cultures (~4 and 3.5 Log_2_FC in mid exponential and stationary phases, respectively), while the expression of the rest of the module was mostly unchanged or downregulated (Supplementary Fig. 4c). On the contrary, the *exoY* gene from the plasmid module was not upregulated in co-cultures, suggesting different regulation from that of the chromosomal module. The *exoY* gene product is the initiator of EPS biosynthesis, and was shown to be a primary target of regulation in *S. melioti* succinoglycan biosynthesis^44,45^. The expression patterns we observed suggest a similar central role of *exoY* in *P. inhibens*. Taken together, *P. inhibens* appears to activate a specific EPS biosynthetic pathway in the presence of algae, and the chromosomal *exoY* is a potential marker gene to follow the production of this *P. inhibens* EPS.

### The *exoY* gene plays a role in bacterial attachment

**Fig. 3:**
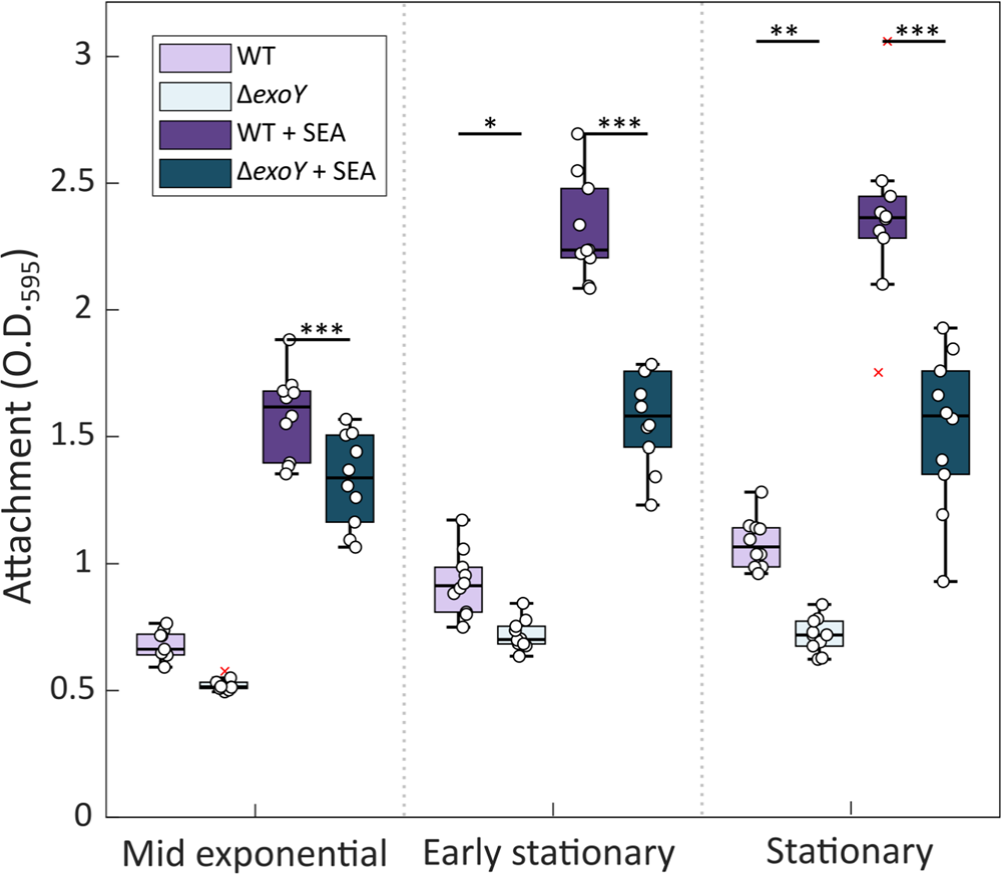
*P. inhibens* attachment capabilities are reduced in *exoY* deletion mutants. Attachment of *P. inhibens* WT (purple) or Δ*exoY* (teal) bacteria was evaluated using an attachment assay (see Material and Methods)^25^. Bacteria were grown in ASW medium supplemented with SEA mix (1 mM) or without added compounds as control. Attachment was assessed by extracting the crystal violet dye from bacterial cells strongly attached to 96-well plates and measuring absorbance at O.D._595_. Bacteria at different growth phases (mid exponential, early stationary, and stationary) were assessed. Each box consists of *n* = 10 wells. Box-plot elements are: center line—median; box limits—upper and lower quartiles; whiskers—min and max values, red x—outlier. Statistical significance was determined using ANOVA, followed by post-hoc Bonferroni test. Asterisks mark treatments in which significantly different attachment was observed between WT and Δ*exoY* bacteria at the same time point for the same treatment. One, two or three asterisks denote p-values lower than 0.05, 0.01, and 0.001, respectively.

The upregulated expression of the chromosomal *exoY* upon SEA mix treatment (Fig. 2) and its high expression in co-cultures (Supplementary Fig. 4b) point to a role of this gene in the production of a specific EPS potentially related to attachment and aggregation of *P. inhibens* bacteria. Therefore, a knockout mutant Δ*exoY*, carrying a deletion in the chromosomal *exoY* copy, was generated and the attachment capabilities to a 96-well plate in response to SEA mix were assessed. The Δ*exoY* bacteria showed reduced attachment in both SEA- treated and untreated cells as compared to wild-type bacteria (WT) (Fig. 3). These results suggest that the chromosomally encoded EPS plays a role in bacterial attachment but appears to function in concert with other attachment mechanisms, as *exoY* deletion reduced but did not abolish attachment. Importantly, growth curves of Δ*exoY* bacteria were similar to WT, both with SEA mix treatment and with no treatment (Supplementary Fig. 5). These observations indicate that *exoY* deletion did not impair the ability of bacteria to respond to the compounds in the SEA mix.

### The synthetic algal exudate mixture promotes bacterial organization in multidimensional structures

While surface attachment marks the initial and essential step in the development of multidimensional structures, the formation of an ECM that supports these structures determines whether bacteria remain as a monolayer or develop into a biofilm^12^. To assess whether conditions that promote attachment of *P. inhibens* bacteria in a 96-well plate, also induce multidimensional organization, we monitored the spatial organization of stationary phase WT or Δ*exoY* bacteria that attached on a glass slide. The effect of the SEA mix addition to bacteria on the thickness of the formed multidimensional structures was visualized using confocal microscopy (Supplementary Fig. 6).

**Fig. 4:**
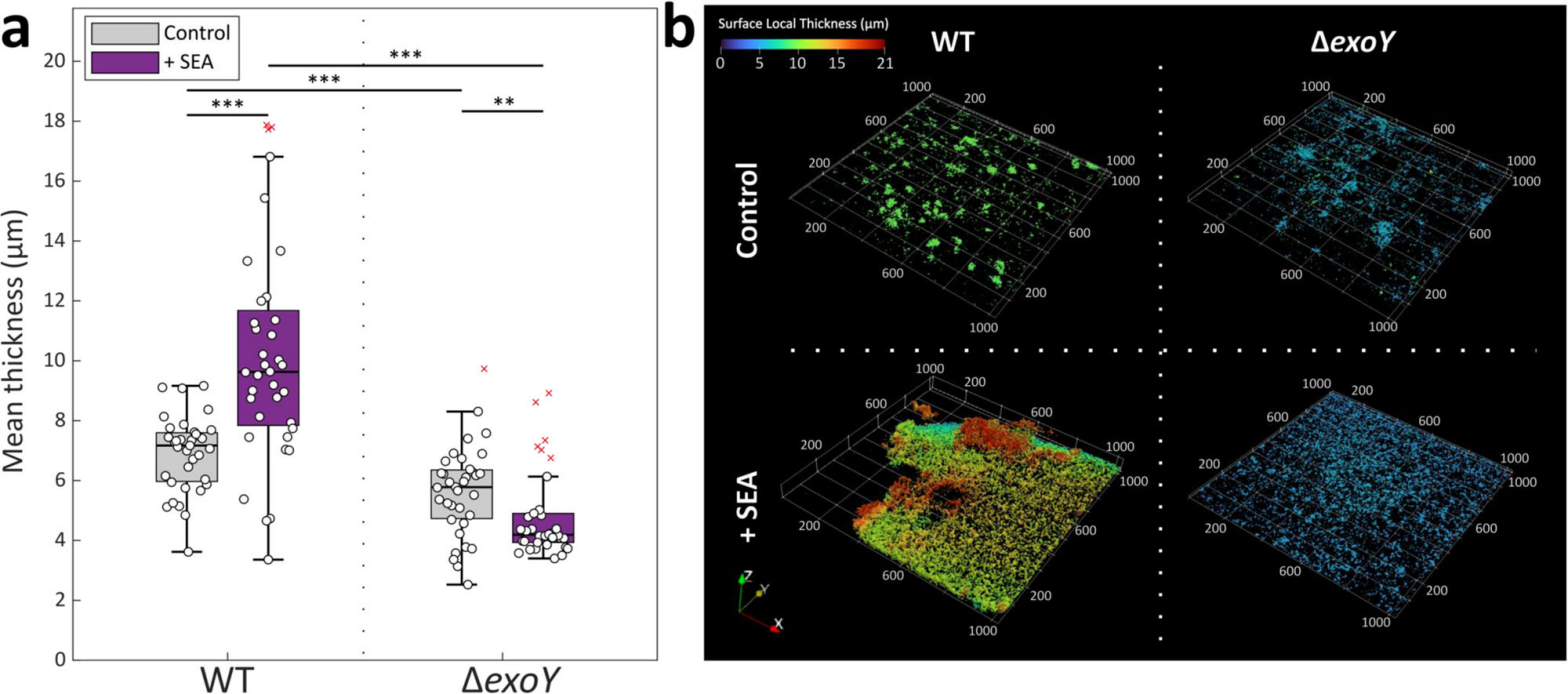
The SEA mix promotes bacterial organization in multidimensional structures. Multidimensional organization of *P. inhibens* bacteria (WT or Δ*exoY*) attached on glass slides was assessed upon treatment with 1 mM of the SEA mix, or a corresponding volume of ASW medium as control. The bacterial structures formed were monitored using confocal imaging of Syto9-stained bacteria at stationary phase, and the thickness was quantified using BiofilmQ^68^ with z-stacks of the imaged slides (see Materials and Methods). **a** Calculation of the mean thickness (µm) was conducted using *n* > 6 random points on five different slides. Box-plot elements are: center line—median; box limits—upper and lower quartiles; whiskers—min and max values, red x—outlier. Statistical significance was determined using Kruxal-Wallis non-parametric test followed by post-hoc Dunn’s test. Two or three asterisks mark statistically different results (*p*-value lower than 0.01 and 0.001, respectively) between the two groups compared. **b** Example of multidimensional structures formed on glass slides by WT (left) or Δ*exoY* (right) bacteria at stationary phase, supplemented with ASW medium as control (top) or SEA mix (bottom). The color bar corresponds to the surface local thickness (µm) used to calculate the mean thickness of each image. X and Y coordinates are shown in µm.

SEA-treated WT bacteria appeared to be organized in a continuous layer (Fig. 4b), exhibiting significantly higher mean thickness of up to 18 μm (Fig. 4a), while untreated WT bacteria attached in thin monolayers with dispersed bacterial patches on the glass surface (Fig. 4b). Mutant Δ*exoY* bacteria, on the contrary, appeared as sparse, small aggregates, with reduced local thickness that was not affected by SEA treatment. Thus, our results demonstrate that the SEA treatment promotes *P. inhibens* organization in multidimensional structures, pointing towards a role of the chromosomally encoded EPS as a scaffold material that supports these structures.

### The synthetic algal exudate array influences bacterial production of polysaccharides involved in attachment and ECM

Our observations suggest that bacterial EPSs play a role in attachment and ECM production of *P. inhibens* bacteria. To characterize the polysaccharides present in the extracellular milieu of SEA-treated WT or Δ*exoY* bacteria, we used fluorophore-conjugated lectins to target and visualize specific sugar monomers. We first targeted the polar polysaccharide of *P. inhibens* bacteria, which has been previously shown to be involved in attachment between bacterial cells and abiotic surfaces^21^. Bacteria grown in liquid medium were stained with a fluorophore-conjugated Wheat Germ Agglutinin (WGA), which binds to N-Acetyl-D-glucosamine (GlcNAc) residues found in the polar polysaccharide.

**Fig. 5:**
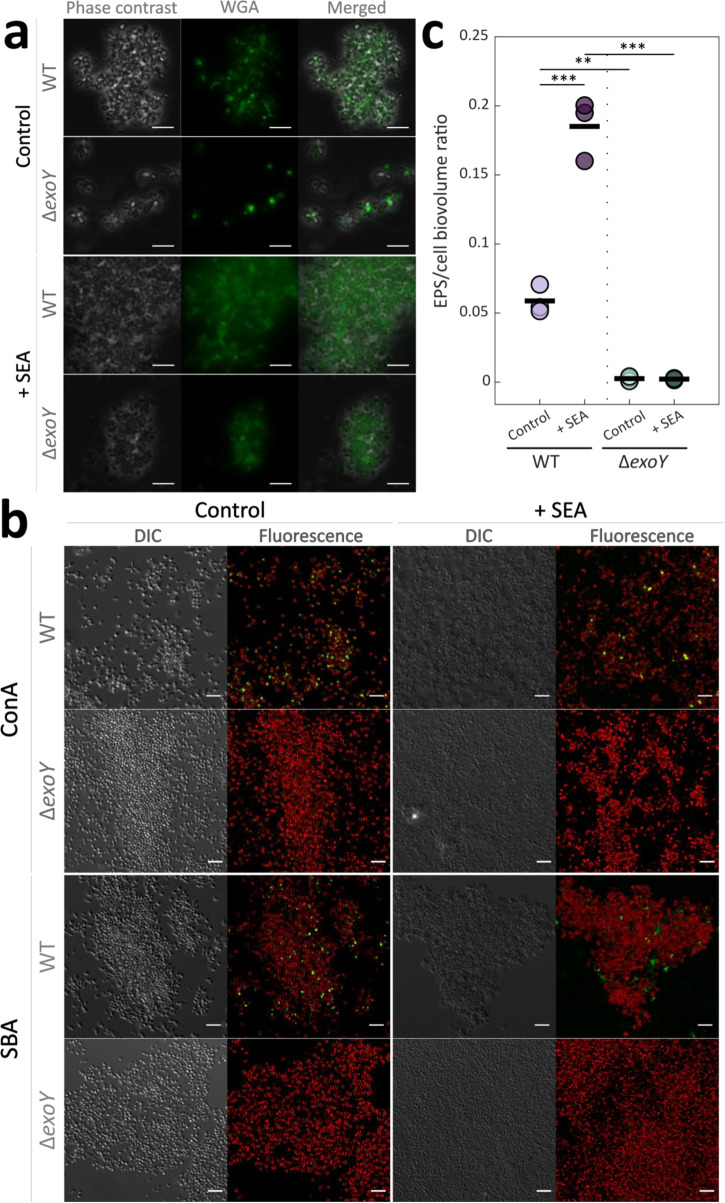
The SEA mix affects the *P. inhibens* EPS. **a** Fluorescence microscopy was used to study the effect of the SEA mix (1 mM) on the *P. inhibens* WT or Δ*exoY* polar polysaccharide. Bacteria were grown to stationary phase in liquid medium and stained with the fluorescent FITC-conjugated Wheat Germ Agglutinin (WGA) lectin, which binds to GlcNAc residues in the bacterial polar polysaccharide. Shown are phase contrast images for general cell abundance, fluorescence of the bound FITC-WGA (green) and the overlay. Scale bars correspond to 5 µm. **b** Confocal microscopy was used to study the sugar composition of the ECM of *P. inhibens*, using lectins to detect specific sugar moieties. *P. inhibens* WT or Δ*exoY* were treated with SEA mix (right) or untreated (left), and grown to late stationary phase on glass slides. The cultures were stained with the FITC-conjugated lectins Concanavalin A (ConA) and Soybean Agglutinin (SBA) that bind glucose and galactose residues, respectively. Shown are DIC (Differential Interference Contrast) images for general cell abundance, the fluorescence of the bound FITC-conjugated lectins (green) and the membrane dye FM4-64 (red) that demarcates bacterial cells. Scale bar corresponds to 5 µm. **c** Quantification of the biovolume of the EPS bound by the lectin SBA, normalized to the biovolume of the cells measured by FM4-64 staining. The biovolumes were calculated using BiofilmQ^68^ based on z-stack images acquired by confocal microscopy, of stationary phase bacterial cultures (WT or Δ*exoY*) grown on glass slides and treated with 1 mM of the SEA mix or untreated as a control. The circles correspond to the EPS/cell biovolume ratio of each image, and the black bars are the mean of *n* = 3 biological replicates. Significance was determined using ANOVA, followed by post-hoc Bonferroni test. Two or three asterisks denote significant differences between the marked groups with *p* values lower than 0.01 and 0.001, respectively.

Bacteria without treatment exhibited the characteristic polar staining as previously described^21^ (Fig. 5a), while SEA-treated bacteria exhibited staining of the entire cell circumference. These observations suggest that the production and cellular arrangement of the bacterial polysaccharide is influenced by the SEA treatment.

Next, we wanted to characterize the *P. inhibens* chromosomally encoded EPS production, by targeting the priming sugar that is added by the *exoY* gene product. Generally, the priming sugars in various wzx/wzy-dependent EPSs are either UDP-glucose or UDP-galactose, and their priming glycotransferases share the same functional annotation (Supplementary Table 2). Aligning *P. inhibens exoY* genes to PANTHER HMM models^46^ revealed that the proteins encoded by the two *exoY* genes are from the family of colanic biosynthesis UDP-glucose lipid carrier transferases (PTHR30576, sub family SF10 for chromosomal *exoY*, and SF4 for plasmid *exoY*). Analysis of the protein sequences and the phylogenetic relationships (Supplementary Fig. 7) of the priming glycotranferases of characterized EPSs from various *Proteobacteria*, supports that both proteins encoded by the *P. inhibens exoY* genes are responsible for priming galactose. Therefore, the *P. inhibens* EPS was targeted by visualizing galactose and glucose residues, using the fluorophore-conjugated lectins Soybean Agglutinin (SBA) and Concanavalin A (ConA), respectively. Importantly, SBA was previously shown to target the galactose residues in the EPS produced by *Rhizobium* species^47^, and EPS deficient mutants also displayed decreased SBA binding^48^. The lectin binding pattern to EPS of WT or Δ*exoY* bacteria was monitored using confocal microscopy of glass-slide attached bacteria at stationary phase, supplemented with SEA mix or untreated (Fig. 5b). The ConA lectin showed similar binding to both SEA-treated and untreated WT bacteria, appearing as small extracellular foci, suggesting that glucose is a common residue in substances secreted by the bacteria. However, SBA binding patterns appeared different between treated and untreated bacteria; in control cultures, binding occurred in small extracellular foci, while SEA-treated WT cultures exhibited a more dispersed signal that seemed to occupy the intercellular space (Fig. 5b and Supplementary Fig. 8). On the contrary, ConA or SBA binding was not detected in the ECM of SEA-treated and untreated Δ*exoY* cultures. Quantification of the biovolume of the EPS bound by the SBA lectin (normalized to the biovolume of bacterial cells), showed significant increase when WT bacteria were exposed to the SEA mix, compared to untreated bacteria (Fig. 5c). However, the biovolume of SBA-bound EPS in Δ*exoY* cultures was significantly lower and did not seem to be affected by the SEA mix treatment. Altogether, these results suggest that the chromosomally encoded EPS is a part of the *P. inhibens* ECM, and the SEA mix promotes its production.

### Bacteria contribute an EPS to form a joint algal-bacterial ECM

The upregulation of the chromosomally encoded *exoY* gene in bacteria treated with SEA mix (Fig. 2) and bacteria in co-cultures with algae (Supplementary Fig. 4c), and the detection of an ECM-related EPS in SEA-treated bacteria, suggest that *P. inhibens* potentially produces this EPS in co-cultures with the algae. In co-cultures, bacteria are commonly found entangled in EPS (Supplementary Fig. 9a), which are often considered to be of algal source^49^. To determine if *P. inhibens* produces EPS in co-cultures, we characterized the sugar composition of the ECMs of algal monocultures and algal-bacterial co-cultures grown in liquid medium. Co-cultures were initiated with either WT or Δ*exoY* bacteria, and growth dynamics were monitored showing comparable dynamics (Supplementary Fig. 10). At mid exponential phase, co-cultures were stained with fluorophore-conjugated lectins and visualized using confocal microscopy.

**Fig. 6:**
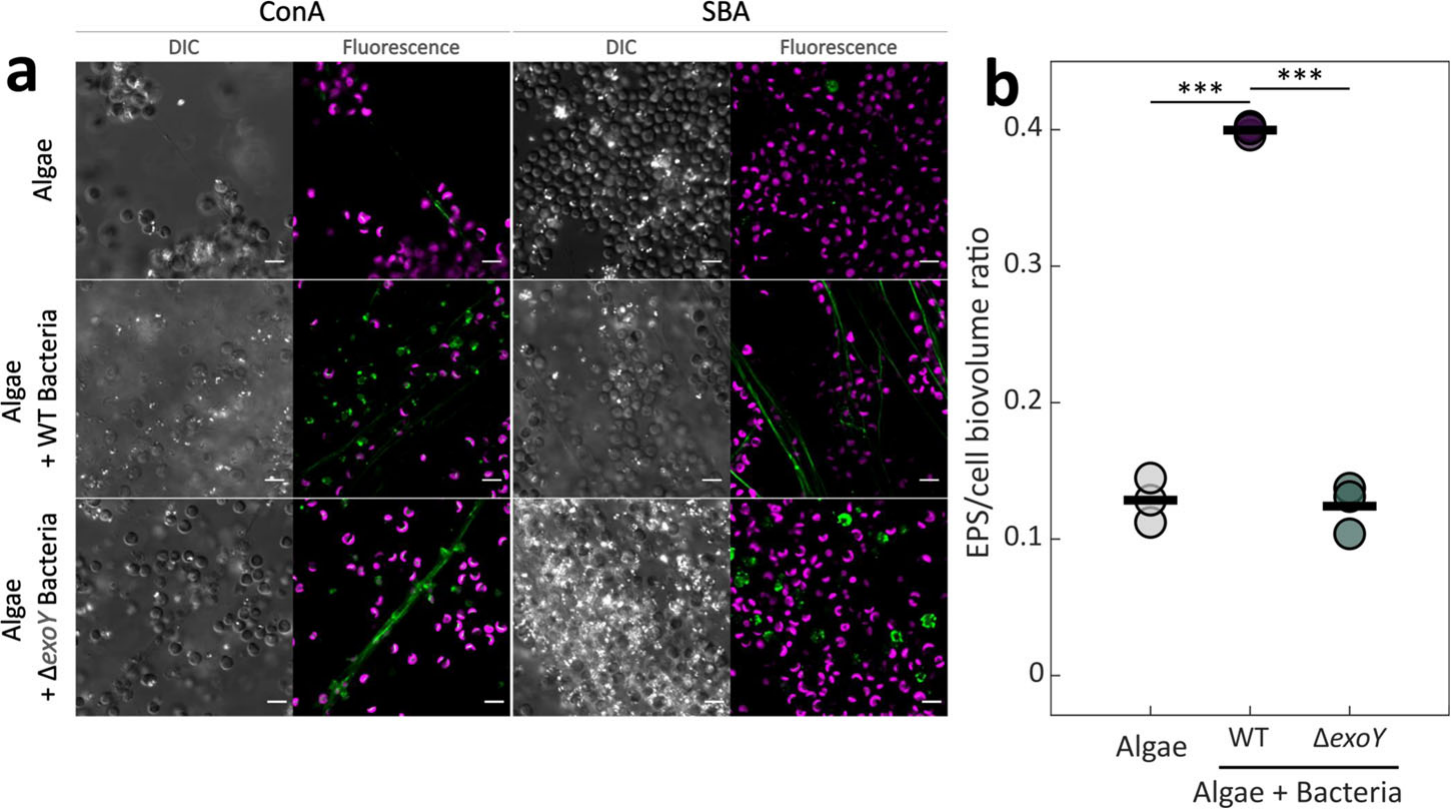
A bacterial EPS is part of the ECM in algal-bacterial co-cultures. **a** Confocal microscopy was used to study the sugar composition of the ECM of *E. huxleyi* monocultures (top), co-cultures with *P. inhibens* WT (middle) or with Δ*exoY* (bottom), using lectins to detect specific sugar moieties. Cultures were grown in liquid medium and imaged at mid exponential phase. The cultures were stained with the FITC-conjugated lectins Concanavalin A (ConA) and Soybean Agglutinin (SBA) that bind glucose and galactose residues, respectively. Shown are DIC (Differential Interference Contrast) images for general cell abundance, the fluorescence of the bound FITC-conjugated lectins (green) and algal autofluorescence of chlorophyll *a* (pink). White particles in the DIC images are algal calcified coccoliths. In this algal strain, only roughly 20% of cells are calcified^88^. Scale bar corresponds to 5 µm. **b** Quantification of the biovolume of the EPS bound by the lectin SBA, normalized to the biovolume of chlorophyll *a*. The biovolumes were calculated using BiofilmQ^68^ based on z-stack images acquired by confocal microscopy, of mid exponential cultures (algal monocultures or co-cultures with WT or Δ*exoY* bacteria) grown in liquid medium. The circles correspond to the EPS/chlorophyll *a* biovolume ratio of each image and the black bars are the mean of *n* = 3 biological replicates. Significance was determined using ANOVA, followed by post-hoc Bonferroni test. Three asterisks denote significant differences between the marked groups with *p* values of lower than 0.001.

Our results indicate that an EPS containing GlcNAc moieties (recognized by WGA) is found in ECMs of algal monocultures and co-cultures with WT bacteria. Thus, GlcNAc-containing EPSs cannot be attributed solely to a bacterial origin (Supplementary Fig. 9b). Additionally, an EPS containing glucose residues (recognized by the ConA lectin) was found in all ECMs, including algal and co-cultures with WT or Δ*exoY* bacteria (Fig. 6a). Therefore, the source of these compounds in the co-culture ECM currently cannot be determined. However, galactose residues were detected by the SBA lectin only in the ECM found in co-cultures with WT bacteria, where it was bound to long extracellular filamentous structures that stretched tens of microns between cells. In contrast, in algal monocultures or co-cultures with Δ*exoY* bacteria, the SBA lectin showed rare binding to foci on algal cells. Furthermore, quantification of the biovolume of the EPS bound by the SBA lectin (normalized to the biovolume of algal Chlorophyll *a*), showed higher amounts of EPS in co-cultures with WT bacteria, than in co-cultures with Δ*exoY* or algal monocultures (Fig. 6b). Altogether, these observations suggest that bacteria produce a specific EPS in response to algal cells, and that both algae and bacteria contribute to the EPS pool to form a joint ECM with a distinct sugar composition.

### Bacterial EPS contributes to algal-bacterial aggregation

Next, we wished to characterize the impact of the bacterial EPS on algal-bacterial aggregation. On a macroscopic level, we observed that algal-bacterial co-cultures show visibly higher aggregation as the cultures age, while algal monocultures of the same age were more homogenous (Supplementary Fig. 11). As we observed bacterial EPS production in co-cultures, we hypothesized that this bacterial EPS could contribute to aggregation in co-cultures. To assess the role of bacterial EPS in the aggregation of algal-bacterial co-cultures, we quantified and compared the number of aggregates in co-cultures with WT versus Δ*exoY* bacteria, or in algal monocultures. Aggregation was quantified by phase contrast imaging of cultures grown in liquid medium at late exponential phase.

**Fig. 7:**
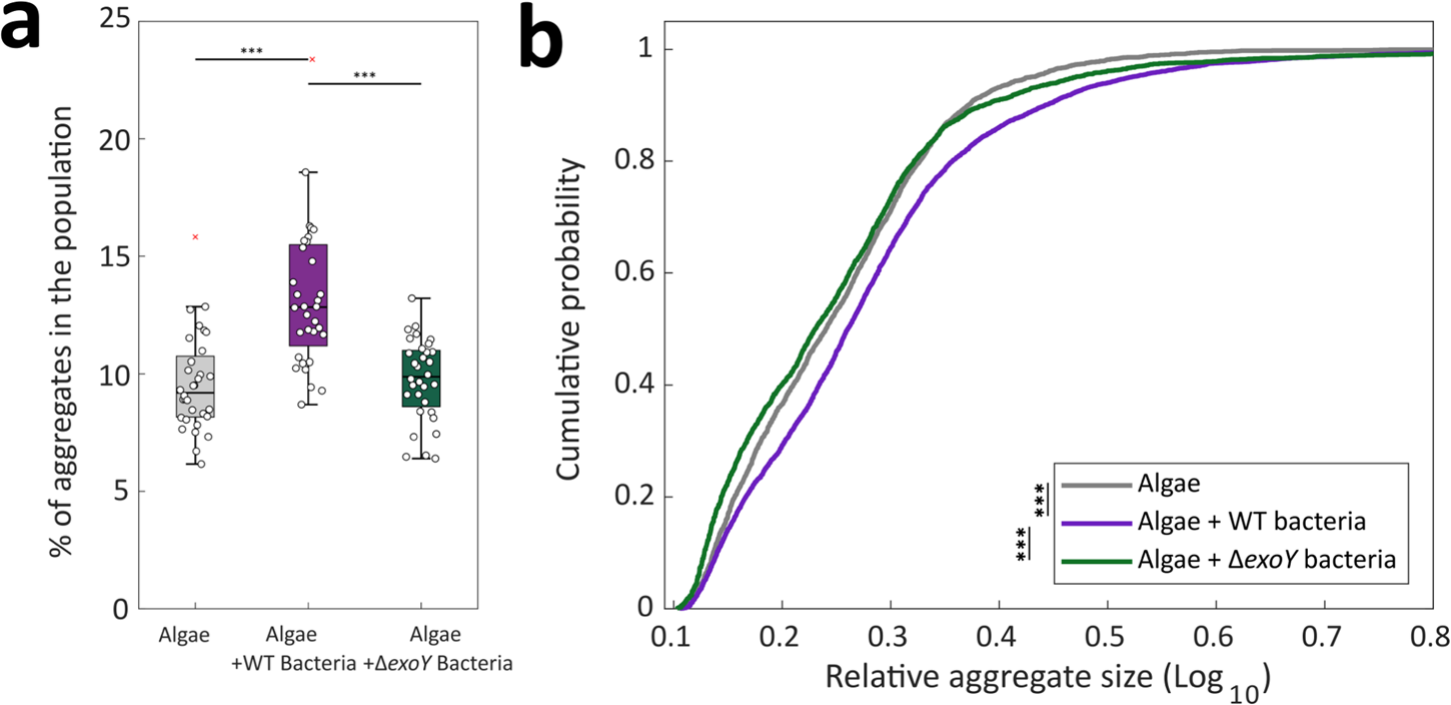
The bacterial chromosomally encoded EPS contributes to aggregation in algal-bacterial co-cultures. Aggregation was measured in monocultures of late exponential algal cells, co-cultures with WT bacteria and with Δ*exoY* bacteria. Aggregation was calculated by imaging algal cells using phase contrast microscopy (see Materials and Methods). Measurements were obtained from *n* = 4 biological replicates, with eight different images for each biological replicate. **a** The percentage of particles considered aggregates in the whole population of particles (single cells and aggregates). Box-plot elements are: center line—median; box limits—upper and lower quartiles; whiskers—min and max values, red x—outlier. Statistical significance was determined using ANOVA, followed by post-hoc least significant difference (Fisher’s LSD) test. **b** Cumulative probability of the relative aggregate sizes (see Materials and Methods). The difference between the cumulative distribution functions was determined using two-sample Kolmogorov-Smirnov tests. Three asterisks denote *p* values lower than 0.001.

Algal-bacterial co-cultures with WT bacteria showed increased number of aggregates compared to algal monocultures, or co-cultures with Δ*exoY* bacteria (Fig. 7a). Additionally, the aggregates produced by co-cultures with Δ*exoY* bacteria were significantly smaller than co-cultures with WT bacteria, and similar in size to aggregates produced by algae grown alone (Fig. 7b). These results suggest that the bacterial EPS functions as an ECM component that agglomerates algal-bacterial structures.

### Genes involved in wzx/wzy-dependent EPS production are expressed in algal-rich environments

**Fig. 8:**
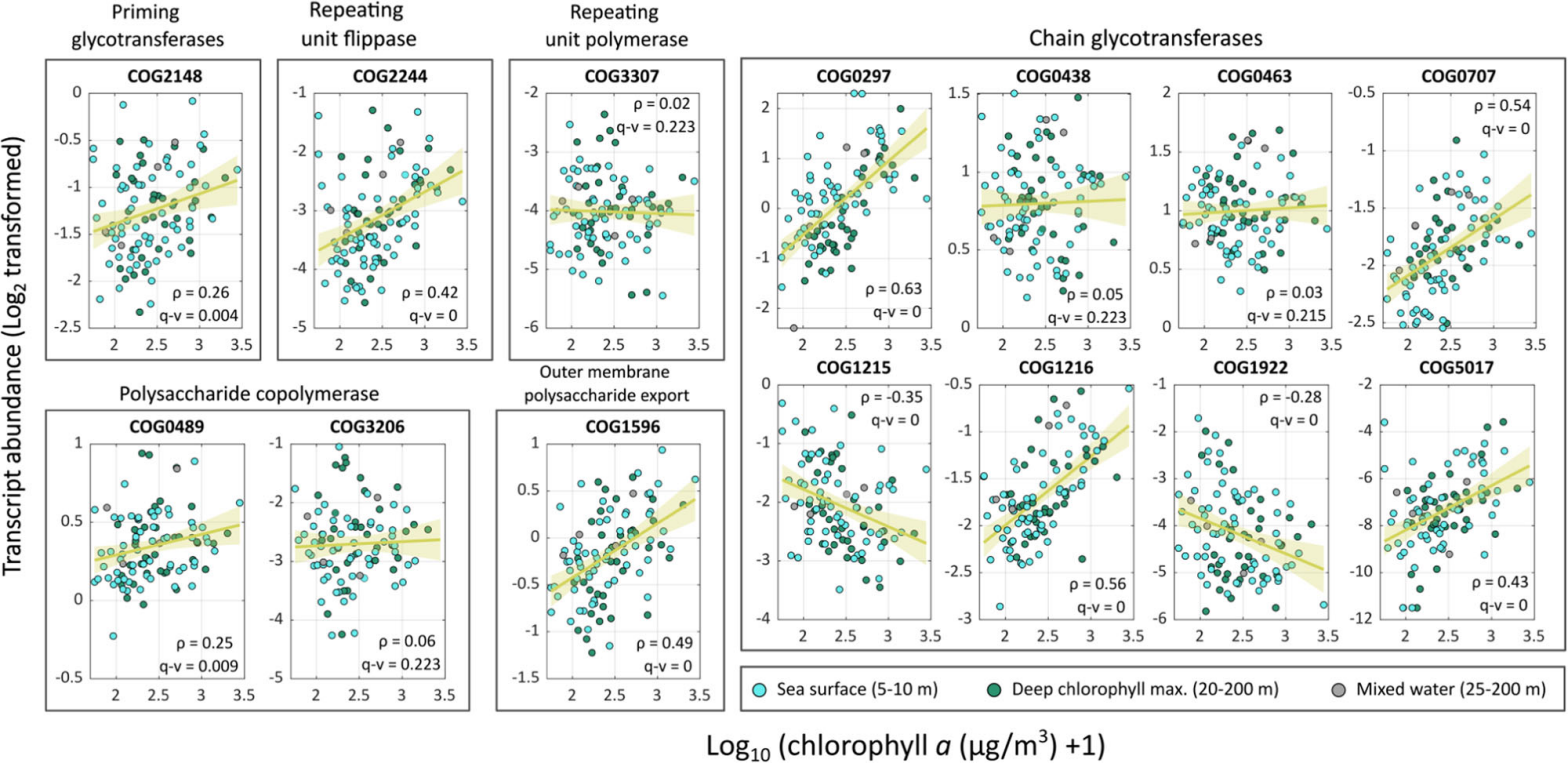
Expression of wzx/wzy- dependent EPS related genes in algal-rich layers of the marine environments. Transcript abundances of orthologous groups (OG)^39^ of genes encoding for proteins involved in wzx/wzy- dependent EPS biosynthesis in the epipelagic region, collected by the TARA expedition^51^. The epipelagic region consists of the sea surface (blue), deep chlorophyll maximum (green) and mixed water layer samples (gray). Transcript abundances (Log_2_ transformed) were fit with a linear regression model (yellow line) and a 95% confidence interval (yellow shade) against the chlorophyll *a* concentrations (Log_10_ (µg/m^3^ + 1) transformed) in the sampling point. Shown for each fit are ρ values calculated by Spearman’s Rank and *q* values corrected with Bonferroni test. Data adapted from ref. ^50^.

Finally, as a first step to explore the significance of bacterial EPSs that are encoded by wzx/wzy modules in environmental algal-bacterial processes, we explored the transcript abundance of key genes in algal-rich environments. To this end, we analyzed oceanic metatranscriptomics^50^ datasets generated by the TARA Oceans expedition^51^. The abundance of transcripts of orthologous groups (OG) of genes involved in wzx/wzy-dependent EPS production was analyzed in the epipelagic layers that are known to inhabit phytoplankton^52^ and are rich in TEP^53^. A moderate yet significant positive correlation was observed between chlorophyll *a* concentration, that is indicative of phytoplankton, and transcript abundance of key wzx/wzy-dependent EPS genes (Fig. 8). Additionally, some chain glycotransferases also showed moderate positive correlation with chlorophyll *a* concentration, while some showed negative correlations, which could be related to variability in the type of the produced EPS. In summary, it appears that transcripts associated with production of specific bacterial wzx/wzy-dependent EPSs are abundant in algal-rich environments, suggesting that bacteria could contribute EPS to the formation of joint algal-bacterial ECM in these environments.

## Discussion

Our data reveals a significant influence of algal exuded molecules on bacterial behavior, facilitating a shift from individual, motile cells to a multicellular lifestyle embedded within an ECM. Previous research has underscored the significance of algal exudates in guiding bacterial chemotaxis towards the phycosphere, highlighting the advantages of this function in actively locating and remaining in this microenvironment^23,54^. Enhanced attachment of *P. inhibens* was previously demonstrated to a membrane separating a bacterial growth chamber from an *E. huxleyi* growth chamber^19^. This increased attachment could be attributed to the bacterial response to algal exudates, which function both as nutrients supporting growth and as chemoattractants facilitating attachment. However, our experimental approach allows the decoupling of these two effects. This is achieved by introducing algal exudates into a medium that is already rich in nutrients, thereby isolating the attachment response from the nutritional aspect. In previous studies^55^, bacterial attachment to TEPs was investigated towards algal TEP that were potentially isolated along with algal exudates. Our research highlights the specific contribution of algal exudates in enhancing bacterial attachment and potentially driving subsequent bacterial EPS production in co-cultures.

Bacterial presence in the phycosphere can be transient^54^, as active mechanisms are required for cells to establish a more enduring connection with a surface^34^. Previous studies have shown that impaired chemotaxis affects bacterial attachment to algal cells or algal-produced TEP^9^, yet the precise impact of algal exudates on the sustained settlement of bacteria in the algal phycosphere was unclear. In our study, we observed that both algal exudates and the SEA mix stimulated bacterial attachment to surfaces (Fig. 1). Moreover, the downregulation of bacterial motility and the upregulation of specific genes potentially involved in EPS biosynthesis upon to SEA treatment or in co-cultures with the algae, suggest a transition from free-swimming cells to the formation of sessile structures^56^. Specifically, the upregulation of the *exoY* gene has been reported previously as a key regulatory event in driving EPS production^44,45^. Similarly, in the present study, upregulation of the *exoY* gene was observed in response to algal exudates coinciding with the formation of multidimensional structures, on both biotic and abiotic surfaces, that are held by a bacterially produced ECM (Fig. 5b). These observations mark the previously unknown contribution of bacterial EPS to the colonization process of the algal environment and suggest a potential commonality in the mechanisms employed by bacteria for attachment to abiotic and biotic surfaces.

Of note, the SEA mixture in the present study is inherently general, representing a broad spectrum of algae. The SEA mix compounds - DMSP, betaine, and succinate - were found in samples from different types of phytoplankton, such as coccolithophores, dinoflagellates, and diatoms, and were noted as significant metabolites produced by algae and utilized by bacteria^24,57^. In contrast, the spent media of *E. huxleyi* contains a chemical fingerprint unique to this specific strain. Consequently, findings derived from the SEA mix treatment likely hold broader applicability to diverse algal-bacterial interactions, whereas results obtained with spent media may be more strain-specific. Additionally, *P. inhibens* bacteria commonly associate with various phytoplankton species including coccolithophores^18^, dinoflagellates^14^ and diatoms^58^, further supporting the broad applicability of our findings. Using the synthetic SEA mix and individual compounds enables the observation of bacterial physiology, such as growth and attachment, in response to particular chemical signals. Although less complex than algal exudates, this approach simplifies the process of gaining mechanistic insights by overcoming the chemical complexity of exudates. Altogether, our findings highlight the multifaceted role of algal exudates, both in simplified and complex arrays, not only in guiding bacterial chemoattraction but also in triggering bacterial processes that promote colonization within the algal microenvironment, in a mechanism potentially similar to biofilm formation.

Findings of the current study demonstrate the interplay of bacterial and algal EPS in algal-bacterial associations. Our investigation reveals a bacterial EPS associated with the ECM in co-cultures, as illustrated in Fig. 6a. This observation indicates the active production of an ECM by *P. inhibens* within the algal environment. Building on previous studies that demonstrated bacterial aggregates forming on algal ECM surfaces^9,55^, we highlight the presence of large, extensively colonized TEPs, referred to as ‘protobiofilms.’ These protobiofilms play a pivotal role in the early stages of biofilm formation, expediting the establishment of biofilms^59^. Our research further demonstrates the production of EPS by *E. huxleyi* in both monocultures (Fig. 5b) and co-cultures with either WT or mutant bacteria (Fig. 6a). This reinforces the existence of an algal-generated matrix, providing a surface for bacterial colonization. Additionally, microscopic observations revealed bacterial cells embedded within TEPs alongside algal cells (Supplementary Fig. 9), suggesting potential attachment of *P. inhibens* to TEPs. The upregulation of a key bacterial gene involved in initiating EPS biosynthesis (Supplementary Fig. 5b) implies that the formation of bacterial ECM is integral to the process of colonizing the algal microenvironment. Interestingly, the EPS observed in co-cultures exhibited a more filamentous appearance (Fig. 6a) compared to bacteria attached to glass, where EPS appeared more amorphous and filled the intercellular space (Fig. 5b). This difference in appearance might arise from the different culture conditions or may be the result of different EPS structures released by bacteria. EPS plays a crucial role in the ECM, holding biofilm structures^12,34^, and its production in co-cultures contributes to the agglomeration of algae and bacteria. Overall, our findings strongly support the hypothesis that both bacteria and algae actively contribute to the EPS pool within a shared ECM.

The collaborative formation of an *E. huxleyi – P. inhibens* ECM facilitates the aggregation of both algae and bacteria (Fig. 7). Algal exudates have been shown to influence the recruitment of certain bacteria and promote symbiotic interactions^24^. The formation of a joint ECM can further support close interactions between algae and bacteria. Previous studies have illustrated that bacteria can establish close proximity through direct attachment to algal cells, playing a crucial role in mediating nutrient exchange^15^. Contrary to direct cell-to-cell attachment, our results demonstrate that *P. inhibens* bacteria can engage in ECM-mediated attachment close to *E. huxleyi* algae, in a process that involves production of bacterial EPS. Since EPS production is considered a costly product^59^, it is tempting to speculate that ECM formation in proximity to algal cells presents benefits for bacteria such as cooperation^60^. One could further hypothesize that the ECM-held algal bacterial arrangement can also benefit algal cells by preventing direct attachment of harmful bacteria. Pathogenic bacteria were previously demonstrated to attach directly to algal cells^61^. Although the protective advantages conferred by the joint ECM to algae remain unexplored, it is plausible that bacteria attached to the ECM are no longer available for direct attachment onto algal cells. This, in turn, could potentially impact their pathogenic capacity.

The present study offers insight into the bacterial contribution to the ECM in response to generic algal compounds, as exemplified by the SEA mixture, and the bacterial reaction to distinct *E. huxleyi*-derived metabolites, as represented by the harvested exudates. Although *E. huxleyi* is a common algal species in the marine environment^62^, its role in environmental mucilage outbreaks remains unknown. Nevertheless, *E. huxleyi* serves as a representative model alga with exudates comparable to those found in various algal species^24^.Uncovering a joint ECM originating from both algae and bacteria invokes important questions about the formation and composition of ECM in marine ecosystems. Our research has revealed that genes related to bacterial EPS are actively transcribed in algal-enriched environments (Fig. 8). However, our understanding of algal-bacterial matrix-building processes in the ocean remains limited. Gaining a deeper understanding of the algal and bacterial roles played in ECM production is crucial for developing strategies to address future challenges in both natural and perturbed marine environments.

## Methods

### Microbial strains and growth conditions

The bacterial strain *Phaeobacter inhibens* DSM 17395 was acquired from the German Collection of Microorganisms and Cell Cultures (DSMZ, Braunschweig, Germany). Bacterial monocultures were cultivated using the following procedure: bacteria were inoculated from a frozen stock (-80°C) onto ½ YTSS agar plates containing 2 g yeast extract, 1.25 g tryptone, and 20 g sea salt per liter (all obtained from Sigma-Aldrich, St. Louis, MO, USA). Plates were incubated at 30 °C for 2 days. Individual colonies were used to initiate bacterial cultures in artificial seawater (ASW) medium. All bacterial monocultures were supplemented with CNPS to support bacterial growth. CNPS consisted of L1-Si medium (details below)^18,63^ supplemented with glucose (5.5 mM), Na_2_SO_4_ (33 mM), NH_4_Cl (5 mM), and KH_2_PO_4_ (2 mM), all sourced from Sigma-Aldrich. The cultures were incubated at 30 °C with continuous shaking at 130 rpm for 2 days. Bacterial concentrations were quantified by measuring OD_600_ values using an Ultrospec 2100 pro spectrophotometer (Biochrom, Cambridge, UK) with plastic cuvettes. Cell numbers were estimated based on OD_600_ values and the cultures were diluted to an initial OD_600_ of 0.01 in ASW medium at the beginning of the experiments involving bacterial monocultures only. Sampling days of bacterial monocultures are referenced as days following the dilution (day 0): mid exponential (1 day), early stationary (2 days), and stationary (3 days).

The axenic algal strain *Emiliania huxleyi* CCMP3266 was obtained from the National Center for Marine Algae and Microbiota (Bigelow Laboratory for Ocean Sciences, Maine, USA). Algae were cultivated in L1 medium based on the protocol by Guillard and Hargraves^64^. Notably, Na_2_SiO_3_ was omitted as per cultivation recommendations for this algal strain, and the medium was designated as L1-Si. Algal cultures were maintained in standing conditions within a growth room at a temperature of 18 °C under a light/dark cycle of 16/8 h. Light intensity during the illuminated period was maintained at 150 mmoles/m2/s. The absence of bacteria in axenic algal cultures was periodically verified through plating on ½ YTSS plates and microscopic observations.

Co-cultures involving *E. huxleyi* and *P. inhibens* were prepared through the following steps: Algal cell concentrations from a late exponential phase culture were enumerated using a CellStream CS-100496 flow cytometer (Merck, Darmstadt, Germany) with 561 nm excitation and 702 nm emission. Each sample involved recording 50,000 events. An inoculum of 10^4^ algal cells was introduced into 30 ml of L1-Si medium and incubated as previously detailed. Following 4 days of algal growth, *P. inhibens* bacteria (prepared as previously described) were quantified using OD_600_ measurements and cell numbers were adjusted to attain a concentration of 10^2^ cells/ml in 30 ml of algal culture, resulting in 10–100 colony forming units (CFU) per ml. Co-cultures were then incubated under conditions identical to algal cultures. Sampling days of algal monocultures or algal-bacterial co-cultures are referenced as days following the addition of bacteria to co-cultures (day 0): early exponential (4 days), mid exponential (7 days), late exponential (9 days), and early stationary (10 days).

### Monitoring bacterial and algal growth

Routine bacterial cell concentrations were determined using optical density measurements at 600 nm (OD_600_) in a spectrophotometer, as detailed earlier. For generating growth curves of bacterial monocultures, bacterial cells were diluted to an initial OD_600_ of 0.01 in 150 µL of the tested medium. The bacterial cultures were incubated at 30 °C in an Infinite 200 Pro M Plex plate reader (Tecan Group Ltd., Männedorf, Switzerland). The incubation process involved alternating cycles of 4 min of shaking and 25 min of incubation, with 300 cycles. Growth was monitored by measuring the absorbance at 600 nm following each shaking step. Growth curves were calculated by subtracting the mean absorbance values of the blanks corresponding to the growth medium. Growth curves were generated using the MATLAB^65^ function shadedErrorBar^66^. Bacterial concentrations in experiments involving co-cultures with the algae were determined using the colony-forming unit (CFU/mL) method. Bacterial cultures were serially diluted in ASW and plated on ½ YTSS plates. The plates were incubated at 30 °C for 2 days, and subsequently, colonies were enumerated to calculate bacterial concentration in CFU/mL. It is important to note that *P. inhibens* rosette formation introduces biases to CFU/mL calculations^21^. Therefore, we compare general growth trends rather than exact cell numbers. Algal cell concentrations in both monocultures and co-cultures were quantified using a flow cytometer, as previously described. To ensure accurate measurements, each algal culture was thoroughly mixed to disperse large aggregates. Subsequently, 100 µL of the prepared samples were transferred to round-bottom 96-well plates. A total of 50,000 events were recorded for each sample to determine algal cell counts.

### Algal spent medium and SEA mix treatment

To prepare algal spent medium, axenic algal cultures were grown as previously described for a duration of 7 days. Subsequently, the cultures were filtered using Thermo Scientific™ Nalgene™ Rapid-Flow™ Disposable Filter Units equipped with a polyethersulfone (PES) membrane of 0.2 μm pore size. This filtration step effectively removed algal cells from the culture. The pH of the resulting filtrate was measured and adjusted to pH 8.0 through careful titration using HCl and NaOH solutions. The SEA mix treatment was formulated to include the following components, each added to the ASW medium, at a final concentration of 1 mM dissolved in MiliQ purified water (unless otherwise stated): DMSP HCl (DMSP, ≥96.0%), NaOH, betaine (≥99%), disodium succinate, all obtained from Sigma-Aldrich, Merck.

### Attachment assays

Bacterial cultures were diluted to an optical density of 0.01 at 600 nm (OD_600_) using 150 µL of the tested medium. The diluted cultures were dispensed into 96-well flat-bottom plates to initiate the assay. To minimize evaporation during incubation, UV-treated Parafilm was gently pressed over the plates to seal each well. The bacterial cultures were incubated at 30 °C with continuous shaking until the assay endpoint was reached. The attachment assay was carried out following established protocols^25^. After incubation, the plates were subjected to the following steps: The culture medium was carefully aspirated, and the wells were rinsed twice with 1× phosphate-buffered saline (PBS, obtained from Sartorius, Beit HaEmek, Israel). This process aimed to remove non-adherent bacterial cells. The plates were dried by exposing them to a temperature of 55 °C for 20 min. The dried plates were submerged in a solution of 0.1% crystal violet. The plates were then incubated at room temperature for 10 min to allow the crystal violet to interact with the attached cells. The staining solution was discarded, and the plates were washed twice with PBS to remove excess crystal violet. The plates were left to dry overnight at room temperature to ensure complete fixation of the stained cells. To quantify the attached cells, the remaining crystal violet was extracted by adding 200 µL of 33% acetic acid to each well. The plates were incubated at room temperature for 15 min to facilitate extraction. Absorbance measurements were conducted at a wavelength of 595 nm using the TECAN microplate reader. The absorbance values were normalized to a blank consisting of the same growth medium, without bacterial cells, that was subjected to the same protocol. Boxplots were plotted using BoxPlotPro^67^ in MATLAB^65^.

### Gene expression analysis in pure bacterial cultures

To assess the expression of genes associated with motility, attachment, and EPS production, bacterial cultures were grown overnight in ASW medium until they reached an optical density of 0.3 (OD_600_). Subsequently, cultures were subjected to the SEA treatment as detailed earlier or left untreated. Following treatment, cultures were incubated for the specified durations, after which bacterial cells were harvested for RNA extraction. A total of 10^8^ bacterial cells were harvested by centrifugation at 4000 rpm for 10 min. RNA was extracted from the harvested cells using the Isolate II RNA Mini Kit (Meridian Bioscience, London, UK), following the manufacturer’s instructions. Cells were disrupted in RLY buffer containing 1% β-mercaptoethanol using bead beating with 100 µm low-binding silica beads (SPEX, Metuchen, Netherlands) at 30 mHz for 5 min. Approximately 1.4 µg of RNA was treated with 4 µl Turbo DNAse (ThermoFisher) in a 50 µl reaction volume. The RNA samples were cleaned and concentrated using the RNA Clean & Concentrator-5 kit (Zymo Research, Irvine, CA, USA) according to the manufacturer’s instructions.

Equal concentrations of RNA were used for cDNA synthesis employing Superscript IV (ThermoFisher), following the manufacturer’s protocols. qPCR was conducted in 384-well plates using the SensiFAST SYBR Lo-ROX Kit (Meridian Bioscience) on the QuantStudio 5 (384-well plate) qPCR cycler (Applied Biosystems, Foster City, CA, USA). The qPCR program encompassed 40 cycles, adhering to the enzyme requirements. The obtained results were analyzed using the QuantStudio 5 software through a relative standard curve approach. Primer efficiencies were determined by performing qPCR amplification on serially diluted genomic DNA, extracted using the Wizard® Genomic DNA Purification Kit (Promega) as per the manufacturer’s instructions. Only primer pairs with at least 80% efficiency were considered. The expression levels of the target genes were normalized using two housekeeping genes: bacterial housekeeping genes *recA* and *gyrA* (Table 1). No DNA contamination was detected when applying the same program to RNA samples that were not reverse-transcribed. Relative gene expression levels were compared to non-treated samples grown under the same conditions.

**Table 1:** Oligonucleotides used for qPCR of *P. inhibens* genes

Gene	Gene ID	Forward Primer	Reverse Primer	Efficiency [%]
*recA*	PGA1_c15260	GCTGACACCCAAGTCGGAG	AGCCGAACATAACGCCAATCT	96
*gyrA*	PGA1_c15140	GCCGATTCCTGACCTCCTTC	TCAGCTTATGTCGGGCTTCG	90
*dltA1*	PGA1_c13760	GTGGATATCACCGAGCAGCA	GGAAAGGCCGCTTTGGTTTT	103
*exoY*_*P*_	PGA1_262p00380	CACACGCGTATCGGAAAGGA	GCAGGAAGAGACCGAGGTTG	106
*exoY*_*C*_	PGA1_c05260	CCCGGTTGGTCGCTTTTTG	GACCTGCCACAATCCGGTAA	100
*exoB*	PGA1_c34470	CTTCCACCTGTGCCACCTAC	CACCCGCCACGTTGAAATAC	98
*motA*	PGA1_c35680	TCCTGGGTGTTTTCCTTGCT	GTGGGATGGGGTGTTCTGAC	107
*motB*	PGA1_c35560	GTCACGGATGAGGGTTTGGT	TATTAGCCACAACGACCGCA	99

### Culture preparation and staining procedures for microscopy

For imaging bacterial biofilms, bacterial cultures were diluted to an optical density of 0.01 at 600 nm (OD_600_) in 12.5 mL of medium. The diluted cultures were grown in Coplin jars containing 7 glass microscopy slides, which had been subjected to dry heat sterilization (170 °C, 3 h). Parafilm was used to secure the lids of the jars, reducing the potential for evaporation. The jars were then incubated at 30 °C for a duration of 3 days to allow for biofilm formation. Prior to staining, the glass slides were washed twice with phosphate-buffered saline (PBS), and excess liquid was removed by gentle tapping. The staining process involved adding 300 µL of the staining solution (described below) onto the cells. Post-staining, the slides were washed twice with PBS to eliminate any unbound dye. A cover slip was placed over the stained cells, and imaging was carried out promptly.

For imaging algal cultures, co-cultures, or non-surface attached bacteria, the following steps were followed: A 1% agarose solution was prepared in ASW. Small agarose pads were created on glass slides, and the sample of interest was gently placed onto these pads. After a 5-min air drying period, the agarose pads were subjected to a gentle wash with PBS to remove any excess liquid. The staining solution, as described below, was applied to the samples on the agarose pads. Following staining, the samples were washed twice with PBS to remove excess dye. A cover slip was carefully positioned over the samples on the agarose pads. Subsequently, immediate imaging was performed.

Alcian Blue staining was performed following established protocols^7^ using a staining solution containing 400 mg L^−1^ of Alcian Blue (8GX, Sigma-Aldrich). The solution was prepared in acidified ultrapure water, adjusting the pH to 2.5 with glacial acetic acid. Samples were incubated with the dye in the dark at room temperature for a period of 2 min. Fluorescent dyes were employed at specific concentrations for labeling: Syto9 (Invitrogen™) - 1.7 µM, FM4-64 (Invitrogen™) - 10 μg/mL, FITC-Labeled Lectins (Vector Laboratories) - 20 μg/mL. Samples designated for fluorescent dye labeling were incubated in the dark at room temperature for a period of 30 min.

### Microscopy and imaging setup

Phase contrast images were captured using a Nikon Eclipse Ni upright microscope fitted with a PLAN 10x Ph1 DL objective lens (Nikon, Tokyo, Japan). Image acquisition was performed utilizing a Nikon DS-Fi3 color camera (Nikon, Tokyo, Japan). For fluorescence and phase contrast imaging, a Nikon Eclipse Ti2-E inverted microscope was employed, featuring a Plan Apo λ 100x Oil Ph3 DM objective lens (Nikon, Tokyo, Japan). Images were acquired using an Andor Zyla VSC-8969 camera controlled by Nis Elements software. Lumencor Spectra X Chroma excitation filters with wavelengths of 470/24 and 575/25 were utilized. FITC-WGA was visualized with an ET519/26 m filter. Laser scanning confocal images were obtained using a Nikon Eclipse Ti2-E A1R HD25 inverted microscope. The system was equipped with PLAN Apo10x λS OFN25 DIC N1 and SR Plan Apo IR AC 60xWI objective lenses (Nikon, Tokyo, Japan). Image acquisition was performed using a Nikon A1 LFOV camera, controlled through Nis Elements software. The scanner selection was Galvano, and the detector selection was DU4 with GaAsP CH2/3 configuration. A first dichroic mirror with wavelengths of 405/488/561/640 was employed. For fluorescence visualization, a 525/50 filter cube was used to capture the fluorescence signal of Syto9 and FITC-labeled lectins. FM4-64 and chlorophyll *a* autofluorescence were captured using 593/46 filter cubes. Consistent image processing protocols were applied across all compared image sets to ensure uniformity.

### Quantification of biofilm thickness

Biofilm thickness was quantified using confocal imaging to visualize Syto9-stained bacteria adhered to glass slides. Z-stack confocal imaging was performed with a step size of 0.5 µm. Initial imaging was conducted at an x10 magnification to identify the attachment zone at the liquid-air interface. Subsequently, images were captured at ×60 magnification from seven randomly selected points for detailed analysis. The BiofilmQ tool^68^ was employed for image analysis. The Syto9-stained images were subjected to segmentation using the Otsu thresholding method. Segmentation utilized a cube with a side length of 20 voxels (equivalent to 5.75 µm). From the segmented images, two key biofilm thickness metrics were calculated: (1) Biofilm Mean Thickness: The average thickness of the biofilm was calculated based on the segmented images. (2) Surface Local Thickness: This metric quantified the local thickness of the biofilm. To represent the multidimensional thickness data, particularly surface local thickness, ParaView^69^, a visualization tool, was utilized. ParaView facilitated the creation of visual representations that effectively highlighted variations in biofilm thickness across the imaged area. Boxplots were plotted using BoxPlotPro^67^ in MATLAB^65^.

### Quantification of the EPS bound by the SBA lectin

The amount of EPS was quantified using confocal imaging to visualize FITC-SBA stained EPS produced by different cultures. Bacterial monocultures were grown on glass slides and stained with FM4-64, as detailed above. Algal monocultures and co-cultures were grown in liquid medium and prepared as detailed above. Of note, large molecules, such as lectins, may not diffuse uniformly into the center of a biofilm^70^, ultimately limiting the ability to image deep into the core of these structures. Additionally, specifically for images of cultures with algae, mixing the cultures can result in clumping. Therefore, mixing was strictly avoided and only fields that exhibit homogeneous staining, and relatively small aggregates, were selected for further analysis. Z-stack confocal imaging was performed with a step size of 0.25 µm and 0.5 µm, for bacterial and algal cultures, respectively. Images were captured at ×60 magnification from three separate slides which represent 3 biological replicates. The BiofilmQ tool^68^ was employed for image analysis. All images were denoised by convolution with kernel size of 5 3 in pixels. Top Hat filter was used with size of 15 voxels (1.59 μm) for both channels of bacteria (FITC and FM4-64), 5 voxels (1.44 μm) and 15 voxels (4.32 μm) for FITC and chlorophyll a channels of algae, respectively. The images were subjected to segmentation using the Otsu thresholding method (3 classes, class 2 is background), with thresholds of 0.085 and 0.07 for FITC and FM4-64 channels of bacteria, respectively, and 0.015 and 0.1 for FITC and chlorophyll a channels of algae, respectively. Segmentation was performed with a cube size of 15 (1.59 μm) and 17 (4.89 μm) voxels side length for bacterial and algal cultures, respectively. Background FITC fluorescence was filtered based on the mean intensity of each cube and only values above 75 and 61.3 A.U. were kept for analysis of bacterial and algal cultures, respectively. The EPS/cell biovolume ratio was calculated as follows:

$$\begin{array}{l}\frac{{EPS}}{{Cell}}{biovolume}\,{ratio}\\=\frac{\sum {Shape}\,{biovolume}\times ({Relative}\,{EPS}\,{abundance}-{Relative}\,{overlape}\,{between}\,{EPS}\,{and}\,{cell}\,{abundance})}{\sum {Shape}\,{biovolume}\times {Relative}\,{cell}\,{abundance}}\end{array}$$

### Microscopy and image analysis for aggregate size distribution

Samples were cultured as previously described. To initiate imaging, 10 µL of each sample was loaded onto a hemocytometer. For every sample, eight outer corner squares (1 mm²) were imaged using phase-contrast microscopy at a magnification of ×10. To capture the complete dimensions of each square, two images were acquired and subsequently manually stitched and cropped using Inkscape (version 1.0.2-2)^71^. This approach ensured uniformly sized images for analysis. FIJI ImageJ^72^ software was employed to calibrate the captured images. For segmentation of cells, the Trainable WEKA segmentation plugin^73^ was utilized. The plugin was trained using a dataset comprising 15 randomly cropped images from diverse samples. Binary segmentation images were generated, which served as the basis for further analysis. Particle analysis was conducted using FIJI ImageJ. Particles with sizes <1 μm² or located on the image border were excluded from analysis. The particle size distribution data, log_10_ transformed, exhibited three distinct parts in the curve. This included a concave curve representing particle sizes smaller than individual cells, a flat curve reflecting the size distribution of single cells, and a convex curve representing the size distribution of aggregates. To extract the size distribution of single cells, linear fittings were applied to the three curve segments (using the findchangepts function in MATLAB^65^). Two significant change points were identified, characterizing the transition points between these segments. Sizes exceeding 3 median absolute deviations (MAD) between these points were classified as aggregates, while sizes less than 3 MAD were categorized as non-cell particles and discarded. Aggregate sizes were normalized by dividing them by the mean of the size distributions of single cells for each respective image.

### Cation exchange resin (CER) extraction of EPS

The EPS extraction procedure using cation exchange resin (CER) was adapted from established protocols^74^. Cultures were grown in 120 mL of the designated medium, as described previously. For the determination of dry weight (DW), a 5 mL aliquot of the sample was subjected to centrifugation at 3200 × *g* and 4 °C for 30 min. The resulting pellet was re-suspended in 1 mL of 1× PBS, transferred to pre-weighed Eppendorf tubes, and then centrifuged again under identical conditions. Following removal of the supernatant, the pellets were dried overnight at 103°C in an oven. The dried pellets were weighed allowing to estimate DW per culture volume for subsequent CER extraction. For the CER extraction, an aliquot of 100 mL of the sample, estimated to contain ~10 mg of DW, was centrifuged at 4000 ×g and 18°C for 15 min. The pellet was re-suspended in 1 mL of 1× PBS, and the same centrifugation conditions were repeated to collect cell aggregates. Subsequently, the pellet was suspended in 1 mL of 1× PBS along with 500 mg of CER beads (Amberlite® IR120 Na^+^ form, Sigma-Aldrich), which corresponded to a ratio of 50 grams of cationic beads per gram of DW biomass. This suspension was incubated for 90 min on a rotary disc shaker at 150 rotations per minute and 4 °C. After the incubation, the suspension was centrifuged (4000 × *g*, 15 min) to isolate the initial supernatant, and the pellet was re-suspended in 1 mL of 1× PBS. Following another round of centrifugation under the same conditions, the two suspensions were combined. The resultant mixture was subjected to dialysis for 2 days using MilliQ purified water and SnakeSkin™ Dialysis tubing (3500 MWCO, Thermo Scientific). The samples were subsequently stored at –80 °C and subjected to freeze-drying (Christ LyoCube, Gamma 2–16 LSCplus) for a period of 3 days to concentrate the samples. For carbohydrate quantification, ~1 mg of the freeze-dried sample was re-suspended in 0.5 mL of 1× PBS. Total carbohydrate content was determined using the Anthrone method, adapted for microplate reading^75^. In triplicates, 50 μL of the sample was mixed with 150 μL of 0.1% Anthrone reagent (ASC, Sigma-Aldrich) dissolved in 96% sulfuric acid (ACS, Sigma-Aldrich). This mixture was incubated in a 96-well microplate at 60 °C for 30 min, followed by cooling to room temperature for 10 min. Polysaccharides were quantified at 620 nm using a TECAN microplate reader. Carbohydrate levels were determined based on a calibration curve derived from glucose standards (ranging from 0 to 100 mg/mL). The amount of carbohydrate per mg of sample DW was calculated using the following equation:

$${Carbohydrates}=\frac{{{Glc}.{eq}.}_{(\mu g)}}{{{DWC}\times ({CM}/{CE})}_{({mg})}}$$

Here, Glc. eq. represents the weight of glucose corresponding to the OD_620_ measurement of the sample, DWC is the dry weight per milliliter of sample, CM is the concentration of the re-suspended sample used for measurements, and CE is the dry weight of extracted EPS per milliliter of sample.

### Functional annotation of genes and phylogenetic analysis

To elucidate the potential functions of genes surrounding the *exoY* genes in *P. inhibens* (PGA1_c05320 on the chromosome and PGA1_262p00380 on the 262 kb plasmid), functional annotation was carried out using EggNOG-mapper v2^39^. This mapping process facilitated the assignment of these genes to specific orthologous groups, providing valuable insights into their potential roles. For protein-coding gene families, the protein sequences of the *P. inhibens exoY* genes were aligned using the PANTHER HMM model sequence alignment tool^46^. This approach aimed to identify aligning gene families and to gain information about potential related functions. A dataset of protein sequences was compiled from the UniProt database^76^, focusing on sequences associated with the KEGG orthologous groups (KO) corresponding to the genes present in the producing bacteria. To ensure specificity, only sequences from the Proteobacteria group were retained for subsequent analysis. To focus on priming glycotransferases, the MAFFT algorithm (version 7.520)^77^ was employed for multiple sequence alignment, utilizing the FFT-NS-2 strategy. The alignment results underwent visual inspection to identify any misalignments or gaps. Notably, no manual editing or refinement was applied, preserving the integrity of the alignment. Aligned protein sequences were utilized to construct a phylogenetic tree, portraying the evolutionary relationships among these sequences. The PhyML software^78^ (version 3.0) was employed for this purpose, utilizing the maximum likelihood method. To enhance accuracy, a Q.pfam +R + F substitution model was chosen, facilitated by Smart Model Selection for PhyML (SMS)^79^. Visualization of the constructed phylogenetic tree was accomplished using the ATGC PRESTO (a Phylogenetic tREe viSualisaTiOn) tool. Subsequent refinement of the tree visualization was conducted manually using Inkscape^71^, optimizing clarity and presentation.

### Transcript abundance analysis in co-cultures

Transcript abundance data for EPS-related genes in *P. inhibens* during co-culturing with *E. huxleyi* were obtained from pre-existing transcriptome data^43^. To enable a meaningful comparison of absolute transcript abundances among the genes within the *P. inhibens* EPS modules, we utilized transcripts per million (TPM) normalization method. These values were generated from bacterial feature counts. This approach not only accounts for variations in gene length but also effectively normalizes the data based on sequencing depth. Evaluation of expression levels of the identified genes within the EPS modules of *P. inhibens* was conducted as follows. *P. inhibens* monocultures grown on glucose were analyzed to establish a baseline for comparison. Then, temporal expression patterns at mid-exponential and stationary phases were analyzed in *E. huxleyi - P. inhibens* co-cultures to assess the algal impact on the bacterial transcriptome during interaction. To identify differentially expressed (DE) genes we utilized DEseq2^80^ (in R version 4.3.1^81^) by comparing samples of bacteria growing with algae to samples of bacteria growing exponentially in pure cultures at two growth phases - mid exponential and stationary. To shrink Log_2_ fold changes (FC) and account for overweighing of features with low counts and high variability we utilized the apglm method^82^. The threshold for DE genes was set to be ±1.5 FC.

### Genetic manipulation of *P. inhibens* bacteria

The deletion mutant Δ*exoY* (strain ES174) was generated through genetic exchange with the gentamycin resistance gene using double homologous recombination^83^. PCR amplifications and restriction-free cloning^84^ were carried out using Phusion High Fidelity DNA polymerase (Thermo Fisher Scientific, Waltham, MA, USA). PCR products were subsequently purified using the NucleoSpin Gel and PCR Clean-up kit (MACHEREY-NAGEL, Düren, Germany). Plasmids were purified using the QIAprep Spin Miniprep Kit (QIAGEN, Hilden, Germany). First, 3 fragments were PCR amplified with ~1000 bp regions upstream and downstream of the target locus. Primers 1 and 2 were used for amplifying the upstream fragment, while primers 3 and 4 were used for the downstream fragment (Table 2). Additionally, a gentamycin resistance gene was amplified from the plasmid pBBR1MCS-5 using primers 7 and 8. The generated PCR products were assembled and cloned into the pCR™8/GW/TOPO™ vector (Invitrogen, Thermo Fisher Scientific, Waltham, MA, USA) using a restriction-free cloning strategy. This resulted in the creation of a knockout (KO) plasmid named pVL1. For transformation, 10 µg of the KO plasmid were mixed with 300 µl of competent *P. inhibens* cells prepared according to established protocols^85^. Competent cells were subjected to electroporation using a pulse of 2.5 kV (MicroPulser, Bio-Rad Laboratories, Hercules, CA, USA), followed by recovery in 2 mL of ½ YTSS medium for 12 h at 30 °C. Transformed cells were then plated on selective ½ YTSS medium agar plates containing 30 µg/ml of gentamycin. Transformants were screened using single colony PCR with primers 5–6 and 9–10. To confirm the desired mutation, individual cell clones were subjected to Sanger sequencing for verification.

**Table 2:** Oligonucleotides used to generate the *exoY*_*C*_ KO mutant

	Primer	Sequence	Reference
1	*exoY*_*C*_ KO upstream forward primer with homology to pCR™8/GW/TOPO™	ACAAAAAAGCAGGCTCCGAATTCGCC CTTAATCACGGCAGTGACATCCGCC	This study
2	*exoY*_*C*_ KO upstream reverse primer with homology to gentamycin cassette	CAGTTTACGAACCGAACAGGCTTATGT CAAATTGGCAACACCCGTCCCAATGAGG	This study
3	*exoY*_*C*_ KO downstream forward primer with homology to gentamycin cassette	GCACTTTGATATCGACCCAAGTACCGCC ACCTAACCGTCCTGGGCTAACTGTGATGG	This study
4	*exoY*_*C*_ KO downstream reverse primer with homology to pCR™II-TOPO™	CTTTGTACAAGAAAGCTGGGTCGAATTCG CCCTCACCATGTCATCGATCACACTGGCG	This study
5	*exoY*_*C*_ verification forward primer	CGCAGGCACTGTGATGGTCC	This study
6	*exoY*_*C*_ verification reverse primer	CGTCGATCAGCTTCTCACAG	This study
7	Gentamycin cassette forward primer	GTTGACATAAGCCTGTTCG	^28^
8	Gentamycin cassette reverse primer	GTTAGGTGGCGGTACTTGG	^28^
9	Gentamycin cassette verification forward primer	GTGCAAGCAGATTACGGTGACG	^28^
10	Gentamycin cassette verification reverse primer	GAGCCTACATGTGCGAATGATGC	^28^

### Transcript abundance of EPS-related genes in marine environments

Transcript abundances of EPS genes were functionally annotated in the current study and documented by ref. ^50^, as part of the TARA Oceans consortium study. Briefly, prokaryote-enriched RNA samples were collected from various ocean layers, and paired-end sequencing was conducted using the HiSeq2000 system (Illumina). Sequencing reads from each RNA sample underwent quality filtering and trimming. These reads were then aligned to the functionally annotated Ocean Microbial Reference Gene Catalog v2. Read counts were normalized based on gene length, summarized according to EggNOG gene families (aligned to the EggNOG version 3 database^86^), and divided by the transcript abundance of a constitutively expressed marker gene. DESeq2^80^ was employed for variance stabilization. The resulting transcript abundances, represented as log_2_-transformed values indicating relative transcript numbers per cell, have been made publicly accessible (https://www.ocean-microbiome.org/). For our analysis, we focused on epipelagic ocean layers, including the surface, mix, and deep chlorophyll maximum layers. In conjunction with the transcript abundance data, we incorporated chlorophyll *a* concentration measurements obtained from the respective samples (10.5281/zenodo.3473199). Using MATLAB^65^, we employed the default linear model (fitlm) to fit the transcript abundances within the context of the available data. This process enabled us to test correlations between transcript abundances of EPS genes and chlorophyll *a* concentration in different ocean layers.

### Statistical analysis

Data analysis and general plotting was done using pre-built functions in MATLAB (R2021b)^65^, unless specified otherwise. All data was analyzed for outliers using the Interquartile Range (1.5 IQR). The distribution of the data points was tested with Shapiro-Wilk test (swtest^87^ function in MATLAB^65^) at *p* > 0.05 cutoff. Leven’s test was conducted to evaluate homogeneity of variance at *p* > 0.05 cutoff. According to the results of these two tests, the specific statistical tests (parametric/non parametric) mentioned in the text were selected. All tests conducted were one sided, shown in figures are relevant *p* values for simplicity.

### Reporting summary

Further information on research design is available in the Nature Research Reporting Summary linked to this article.

### Supplementary information

Supplementary Material

Reporting Summary

## Data Availability

The datasets used and/or analyzed during the current study available from the corresponding author on reasonable request.
