# Supplementary material for: Bacteria contribute exopolysaccharides to an algal-bacterial joint extracellular matrix
Source: NPJ Biofilms Microbiomes. 2024 Apr 1;10:36. doi: 10.1038/s41522-024-00510-y (PMC10984933; doi:10.1038/s41522-024-00510-y)
Supplement: Supplementary file 1 — Supplementary Material [file 41522_2024_510_MOESM1_ESM.pdf]

Supplemental Data

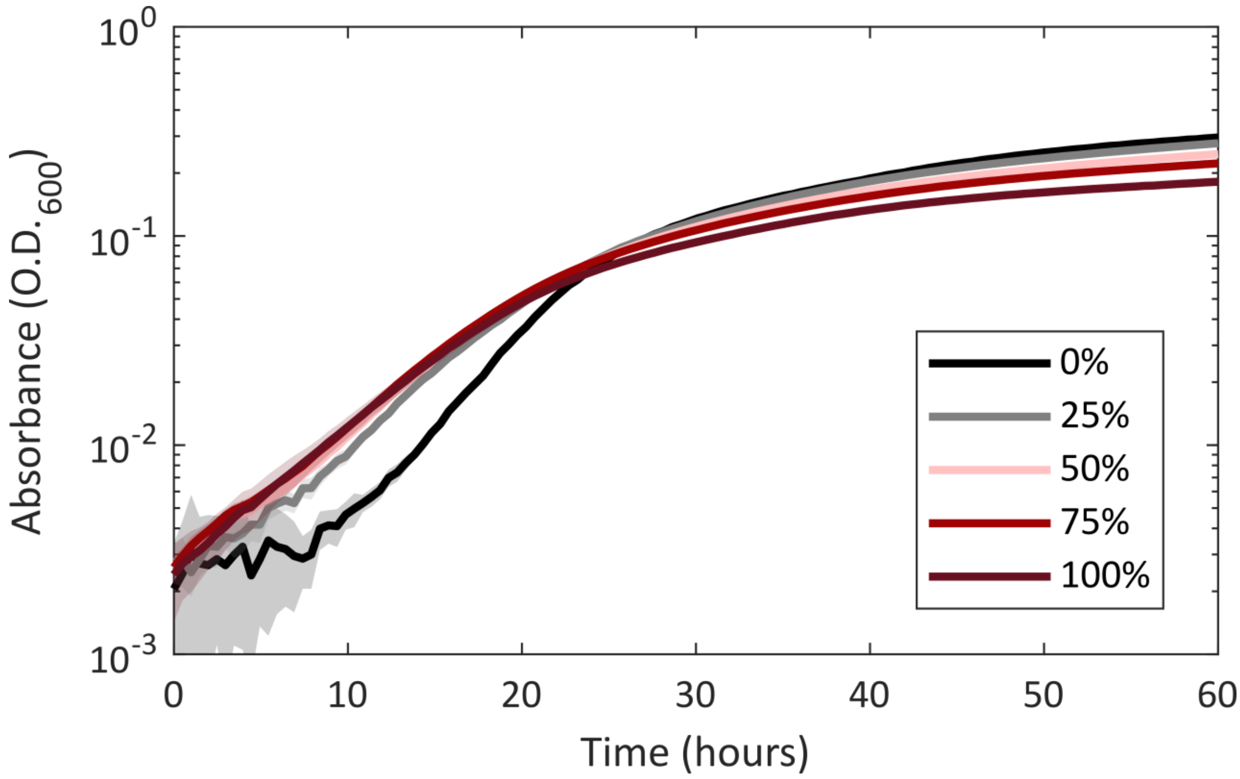

Supplementary Figure 1. Addition of *E. huxleyi* algal spent medium does not increase the maximum yield of *P. inhibens* bacteria. Growth of *P. inhibens* bacteria was measured by $O.D._{600}$ in 0-100% (v/v) concentrations of spent medium extracted from exponentially growing *E. huxleyi* algal cultures, mixed with ASW. Lines represent the mean of n = 6 biological replicates with shaded error bars. All concentrations of spent medium induced lag-phase shortening compared to ASW (0%), as previously described[1]. However, the maximum O.D. was not increased upon treatment with algal spent medium and was even slightly lower at higher spent medium concentrations.

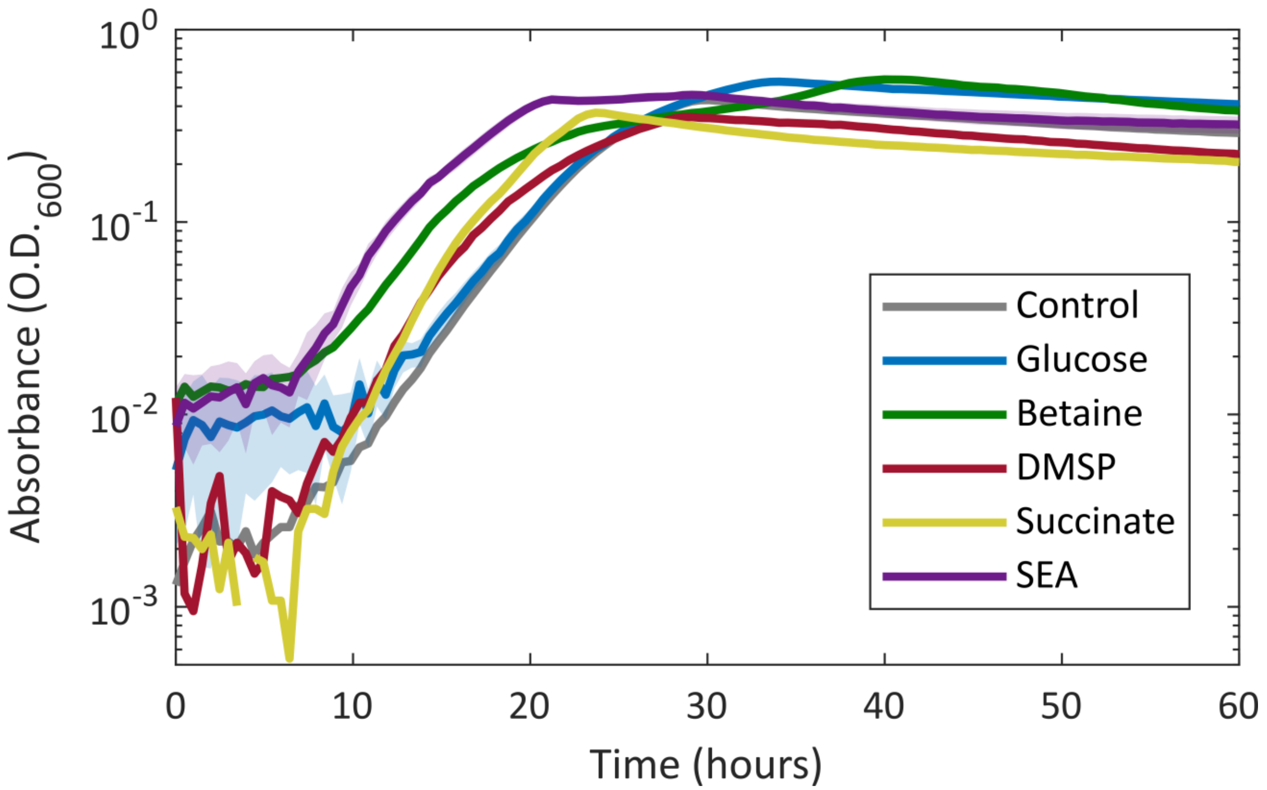

Supplementary Figure 2. Addition of known *E. huxleyi* algal exudates does not increase the maximum yield of *P. inhibens* bacteria. *P. inhibens* bacteria were supplemented with 1 mM of DMSP, betaine, succinate, or a mixture of 1 mM of each compound (the SEA mix). As controls, bacteria were grown without any supplements or with 0.6 mM glucose, to allow comparison with the amount of carbon in succinate. Growth was measured by $O.D._{600}$. Lines represent the mean of n = 6 biological replicates with shaded error bars. DMSP, betaine, succinate and SEA expedited the bacterial lag-phase compared to the control, as previously described[1]. However, the maximum O.D. of bacteria supplemented with SEA, DMSP and succinate was equal or slightly lower than the control.

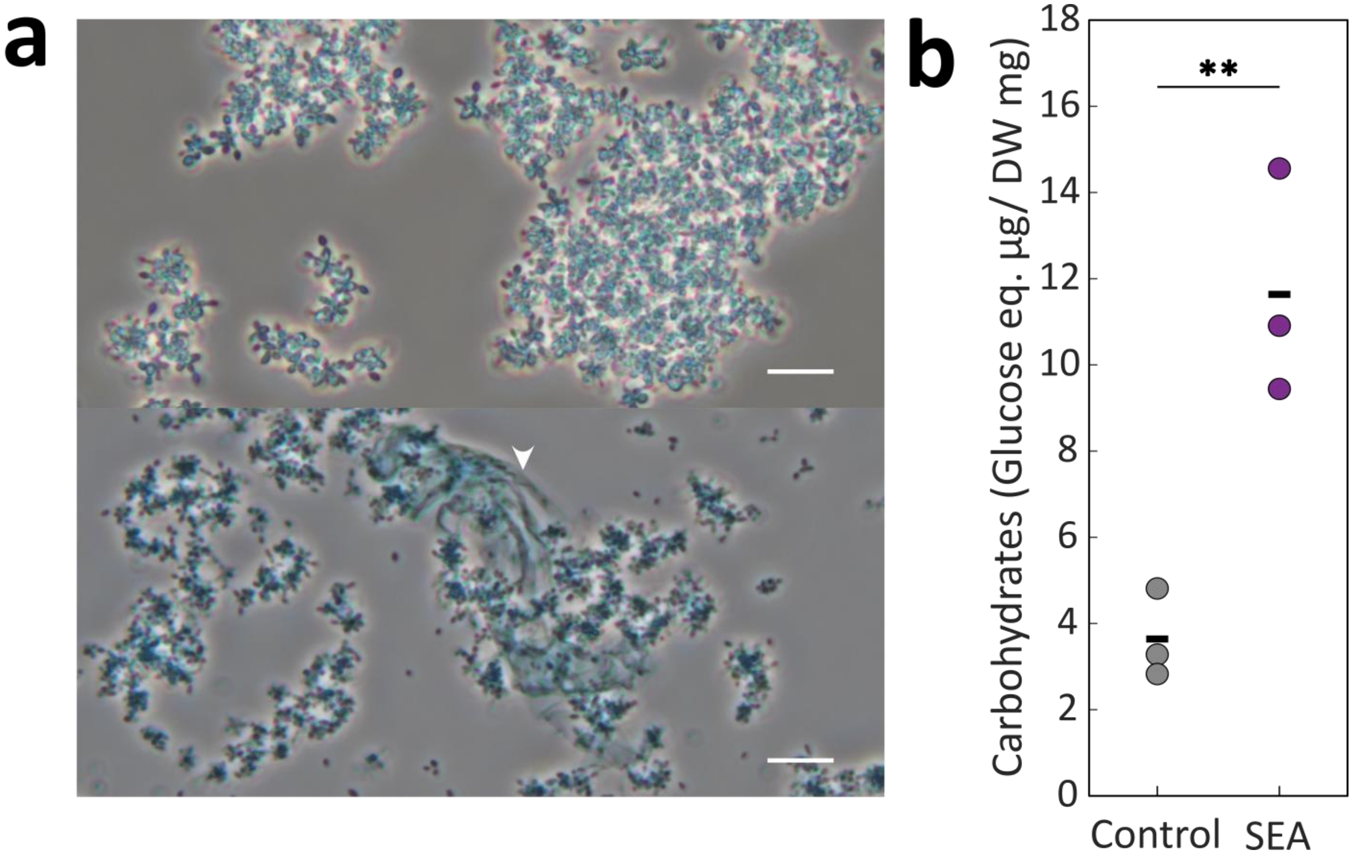

Supplementary Figure 3.The SEA mix promotes bacterial extracellular matrix (ECM) production. (a) Bacterial cultures at early stationary phase were stained with Alcian Blue to detect acidic EPS. SEA-treated bacteria exhibited extracellular sheet-like structures that were stained (bottom, marked by a white arrowheads) while untreated cells did not exhibit similar structures (top). Scale bar represent 20 µm. **(b)** Quantification of extracellular carbohydrates extracted from SEA treated (purple) or untreated (gray) bacteria at stationary phase. The black bars are the mean of the carbohydrates extracted from n = 3 biological replicates. Amount of carbohydrates is represented by the equivalent weight of glucose, normalized to the dry weight (DW) of the sample. Statistical significance was calculated using two sample t-test, two asterisks denote p-value lower than 0.01.

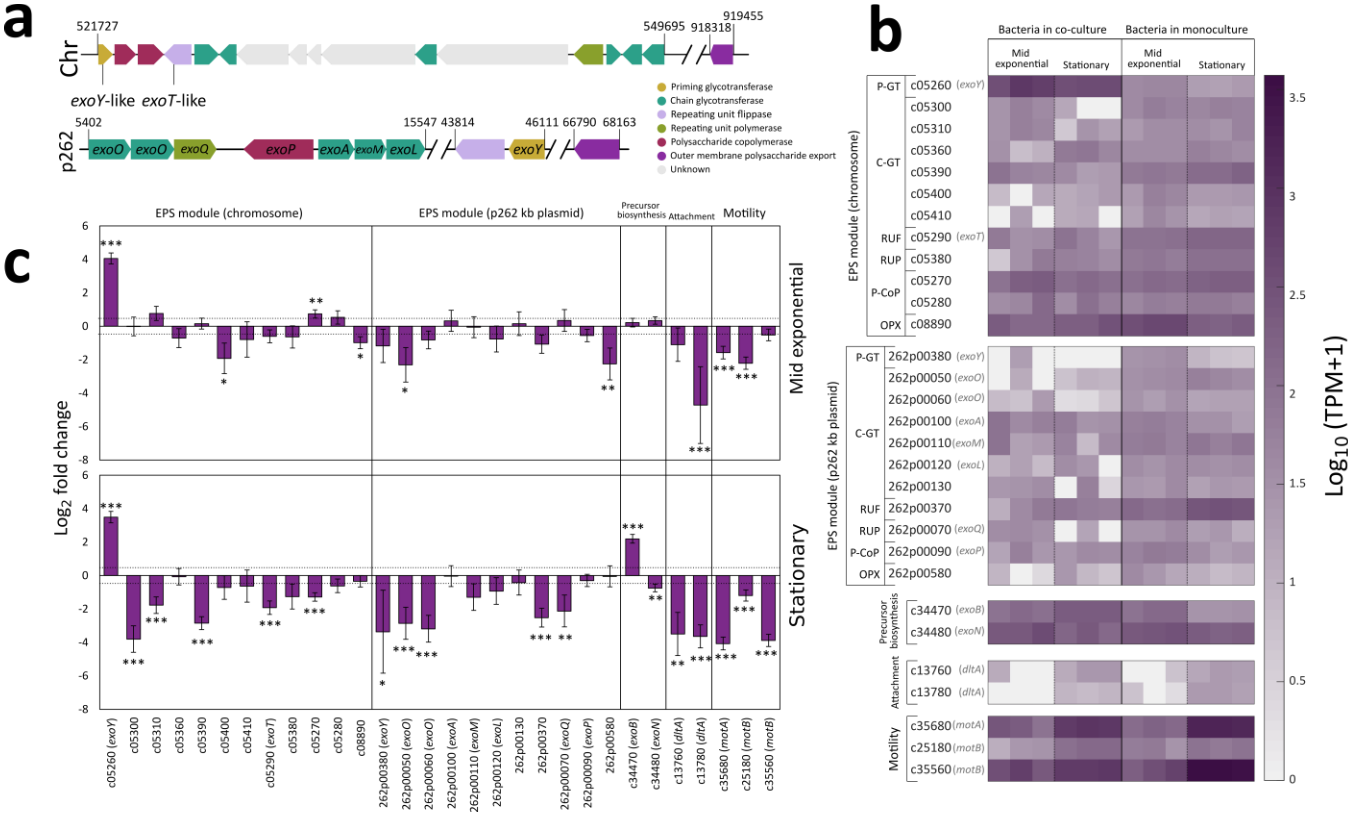

Supplementary Figure 4. ***P. inhibens*** **EPS module expression in co-cultures with** ***E. huxleyi*** **algae. (a)** *P. inhibens* genes encoding for proteins potentially involved in succinoglycan-like EPS production are clustered on the chromosome (top) and the p262 kb native plasmid (bottom). **(b)** Expression of the identified EPS-related genes, in addition to gene markers for motility and attachment, was analyzed in liquid co-cultures of *P. inhibens* bacteria with *E. huxleyi* algae or in bacterial monocultures in mid exponential or stationary phases. For Each gene, the number of transcripts per million bacterial cells (Log_{10}(TPM+1)) was calculated from n = 3 biological replicates. Shown is the functional annotation for each gene (P-GT – priming glycotransferases, C-GT – chain glycotransferases, RUF – repeating unit flippase, RUP – repeating unit polymerase, P-coP – polysaccharide co-polymerase, OPX – outer membrane polysaccharide export) and below the gene entry. **(c)** The Log_2 fold change of the expression of each gene in co-cultures over the expression in bacterial monocultures. Significant differential expression was considered ±1.5 FC and tested using the Wald test as a part of DEseq2[2]. One, two or three asterisks denote adjusted p-values lower than 0.05, 0.01 and 0.001, respectively.

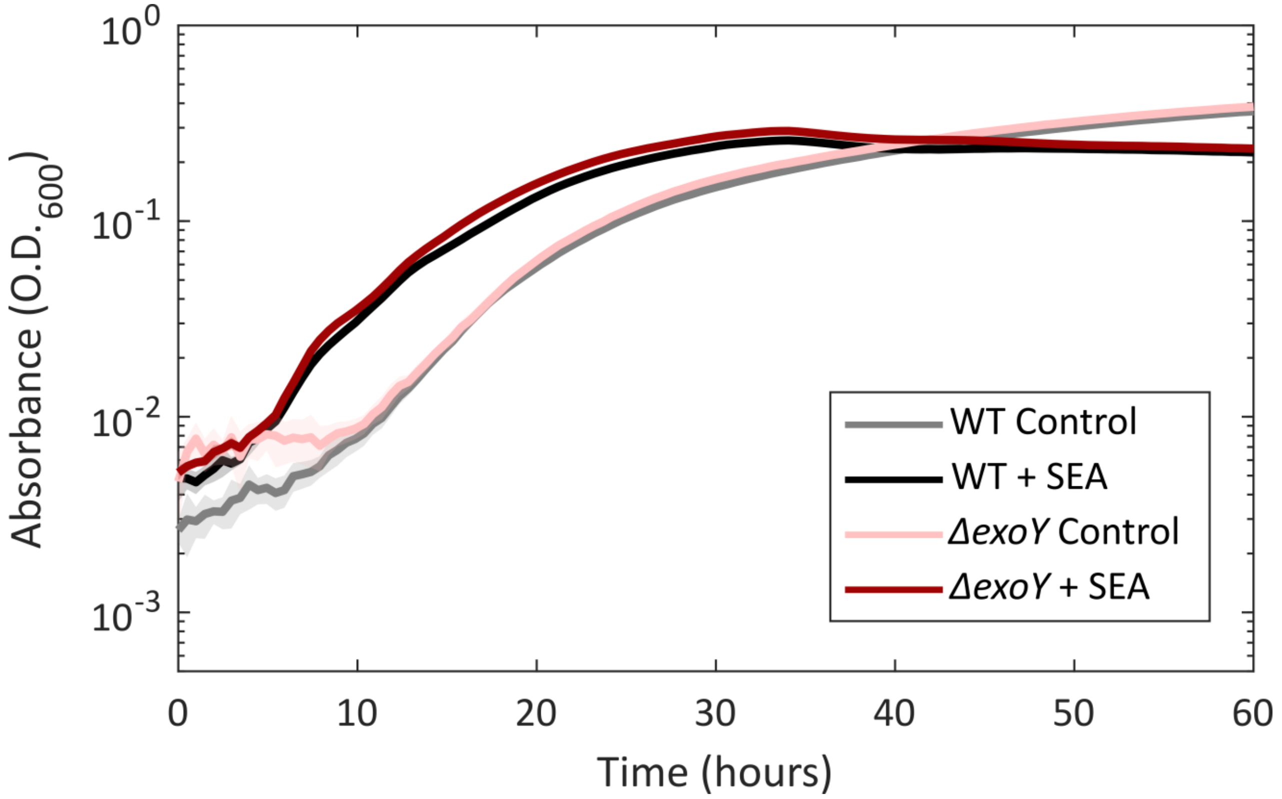

Supplementary Figure 5. Δ*exoY* bacteria exhibit growth dynamics similar to WT bacteria. *P. inhibens* WT or Δ*exoY* bacteria were supplemented with SEA mix (1 mM) or untreated. Growth was measured by OD_{600}. Lines represent the mean of n = 4 biological replicates with shaded error bars. Δ*exoY* mutant showed similar growth to WT both when supplemented with SEA or untreated. both Δ*exoY* and WT bacteria showed shorter lag-phases in response to the SEA treatment, as previously described[1]. Maximum OD reached by Δ*exoY* was similar to WT both for SEA-treated and untreated cultures.

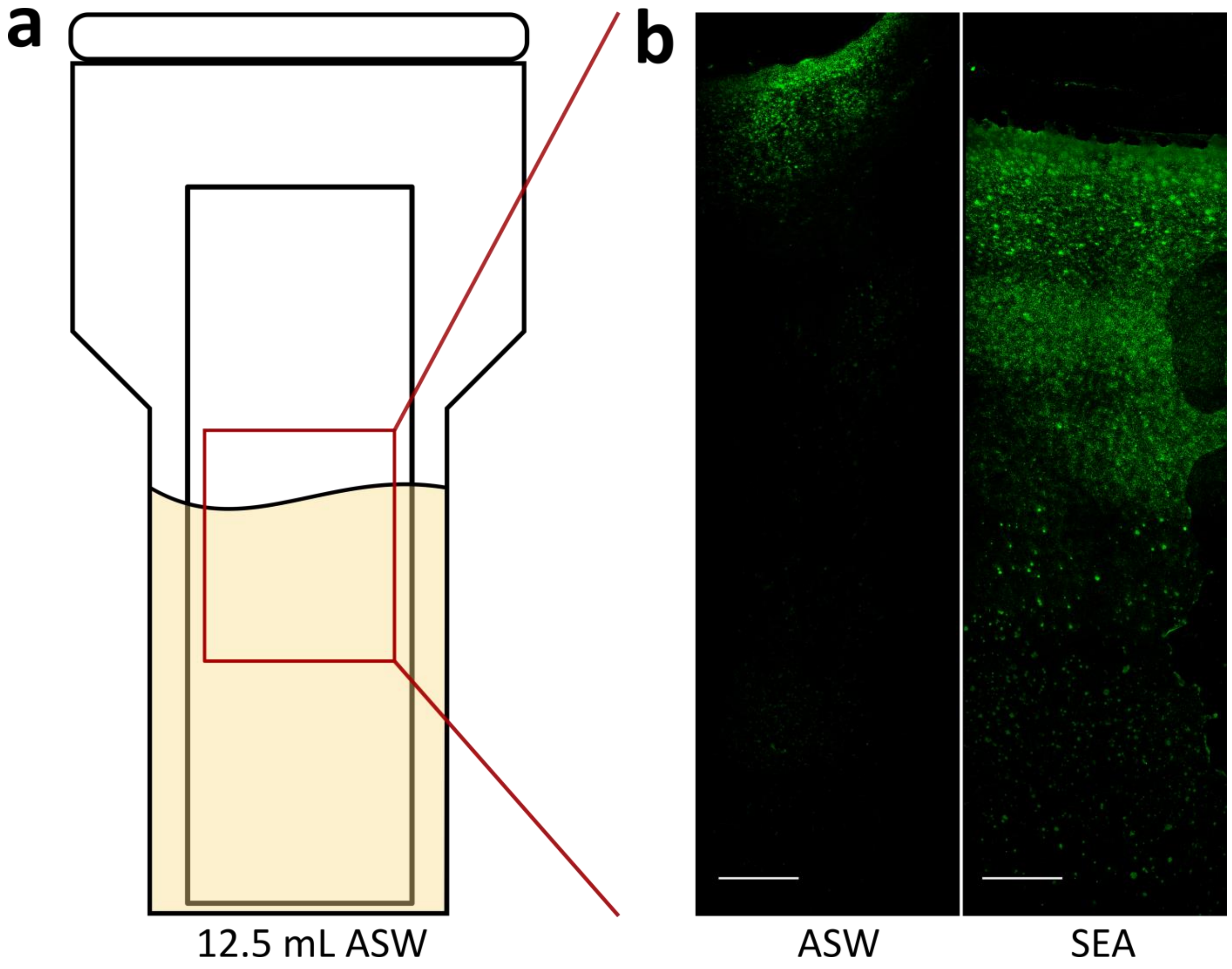

Supplementary Figure 6. SEA treatment increased *P. inhibens* attachment on glass slides. (a) Schematic representation of the experimental setup. *P. inhibens* bacteria were grown in Coplin jars and glass slides were inserted to the jars to serve as a surface for attachment. Bacteria were supplemented with SEA mix (1 mM), or untreated, and grown to stationary phase. **(b)** Representative stitched confocal images of the glass slides (×10 magnification). For visualization and quantification, each slide was stained with Syto9 (green). As can be seen, bacterial structures mainly formed at the liquid-air interface. Scale bars represent 1000 µm.

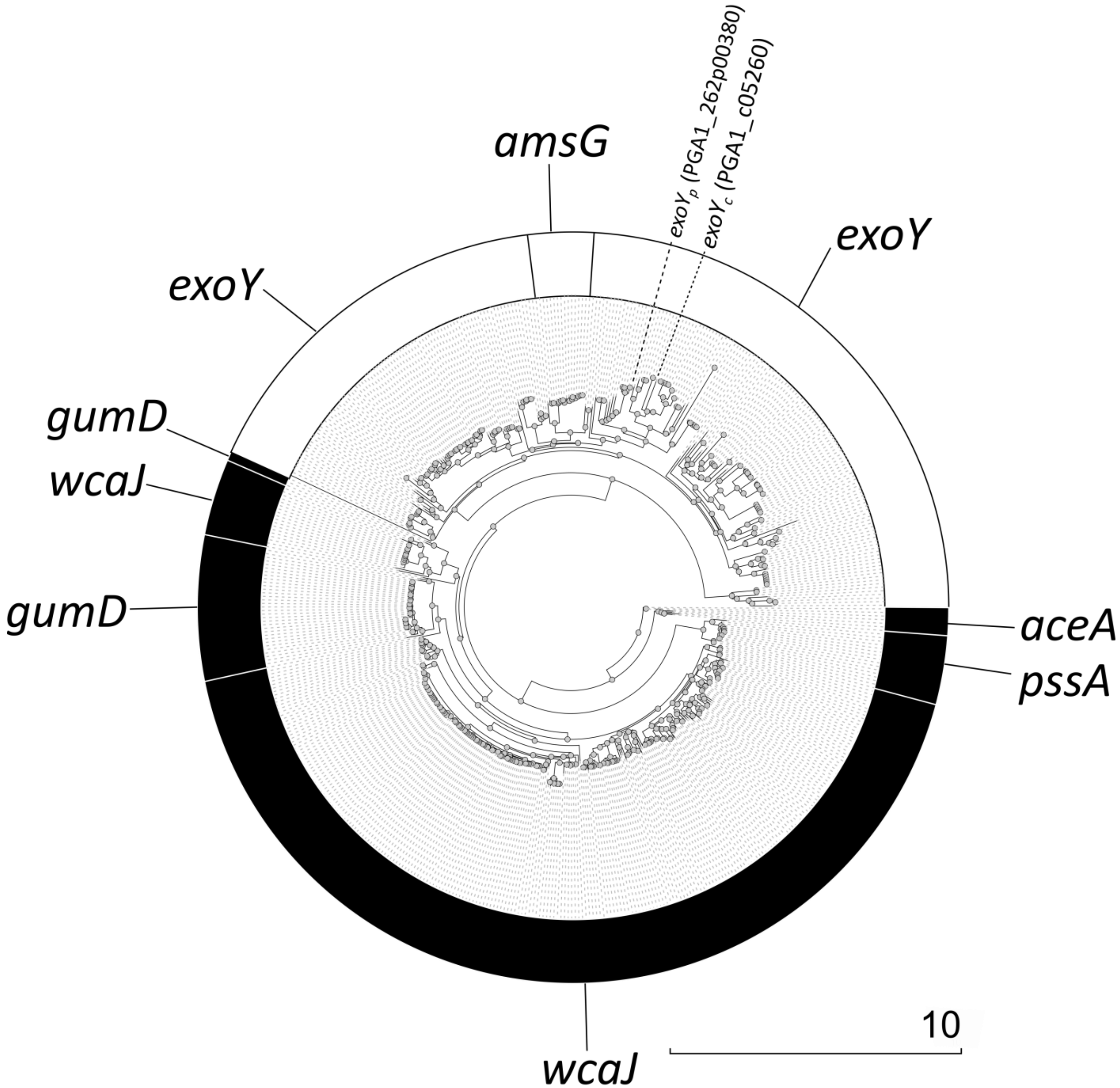

Supplementary Figure 7. Phylogenetic tree of priming glycosyltransferases of different wzx/wzy-dependent EPS. The protein sequences encoded by the genes of the priming glycotransferases of characterized EPS (Table S2) from *Proteobacteria* were used to perform a multiple sequence alignment. A phylogenetic tree was reconstructed to study the clustering of UDP-glucose (black) and UDP-galactose (white) transferases. The two *P. inhibens exoY* genes are marked inside the UDP-galactose transferases clusters.

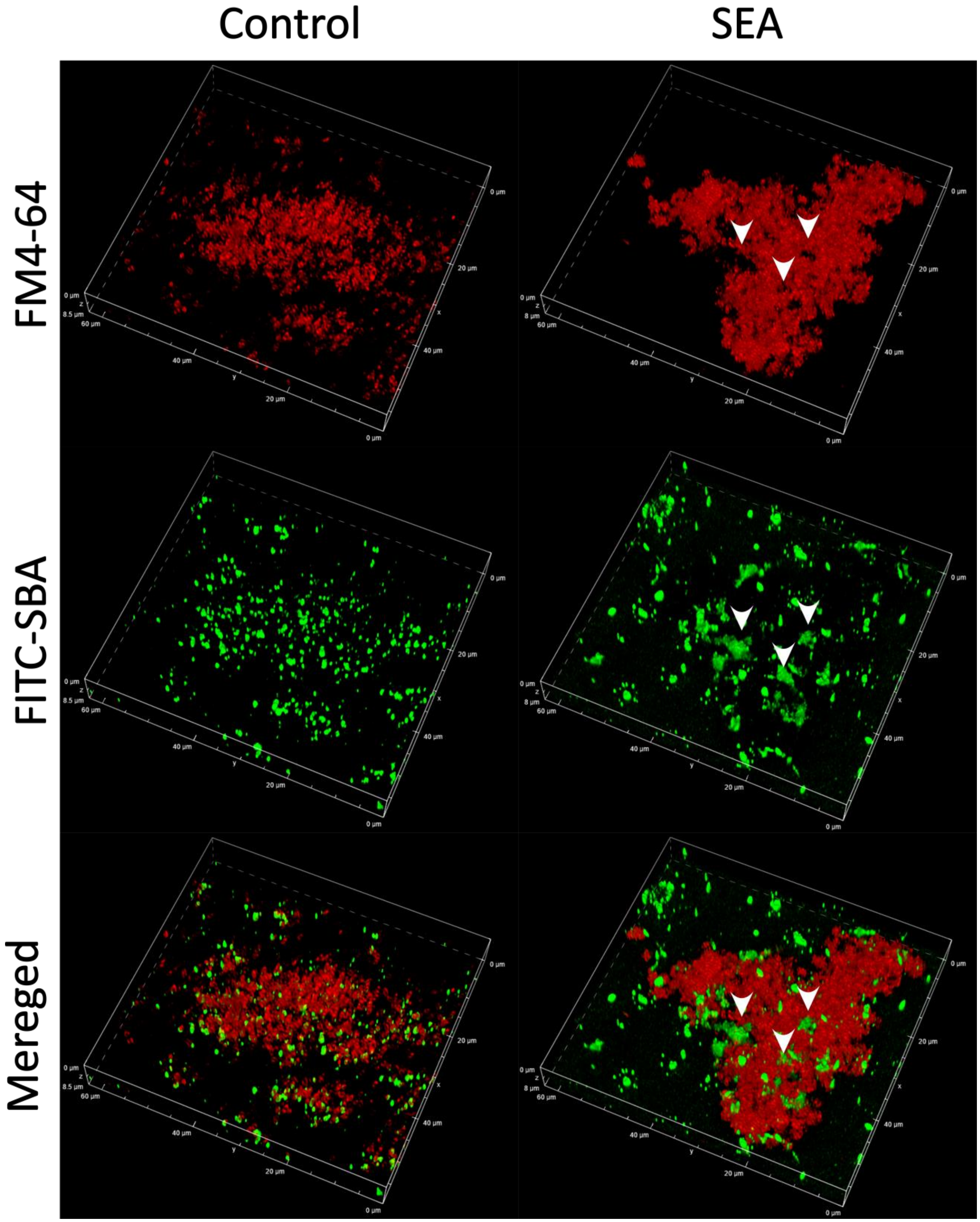

Supplementary Figure 8. The SEA mix affects patterns of SBA lectin binding of *P. inhibens*. Volume configuration of the confocal microscopy image shown for WT bacteria 5B. Bacterial *P. inhibens* monocultures were grown to stationary phase on glass slides and treated with SEA (right) or were untreated (left). Shown are the fluorescence of the bound FITC-conjugated Soybean Agglutinin (SBA, green) that binds galactose residues and the membrane dye FM4-64 (red) that demarcates bacterial cells. White arrowheads show examples of FITC-SBA binding to regions that appear to fill intercellular spaces (see cell distribution according to the membrane stain, FM4-64).

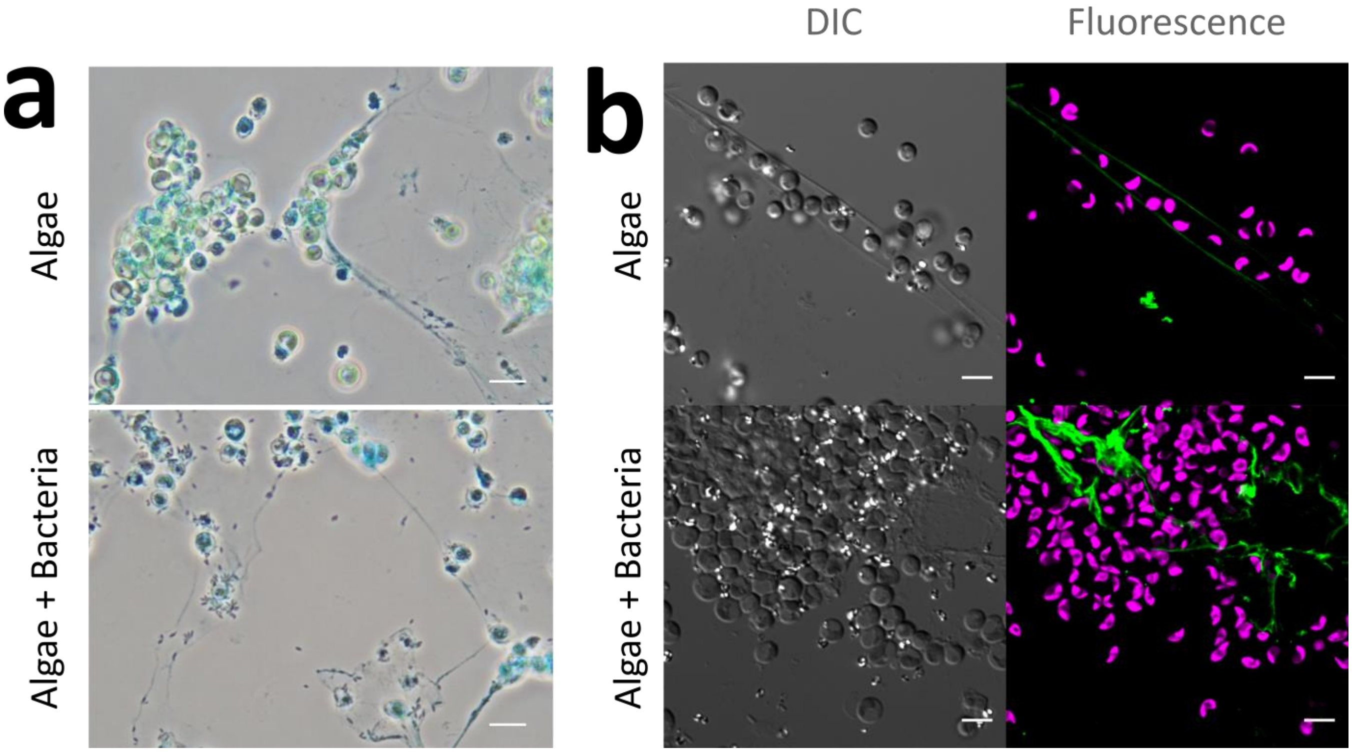

Supplementary Figure 9. Algae and bacteria produce an extracellular matrix (ECM). (a) Algal monocultures and algal - bacterial co-cultures were stained with Alcian Blue to stain extracellular acidic polysaccharides. Stained threads are seen both in cultures of early stationary phase *E. huxleyi* monocultures (top) and *E. huxleyi* - *P. inhibens* co-cultures (bottom). In co-cultures, *P. inhibens* bacteria are seen in close proximities to the threads. Scale bar represent 20 µm. **(b)** Algal monocultures and algal - bacterial co-cultures were stained with FITC-conjugated Wheat Germ Agglutinin (WGA) that targets GlcNac moieties. Stained threads are seen both in cultures of mid exponential phase *E. huxleyi* monocultures (top) and *E. huxleyi* - *P. inhibens* co-cultures (bottom). Shown are DIC (Differential Interference Contrast) images for general cell abundance, the fluorescence of the bound FITC-WGA (green) and algal autofluorescence of chlorophyll *a* (pink). White particles in the DIC images are algal calcified coccoliths. In this algal strain, only roughly 20% of cells are calcified[3] .Scale bar corresponds to 5 µm.

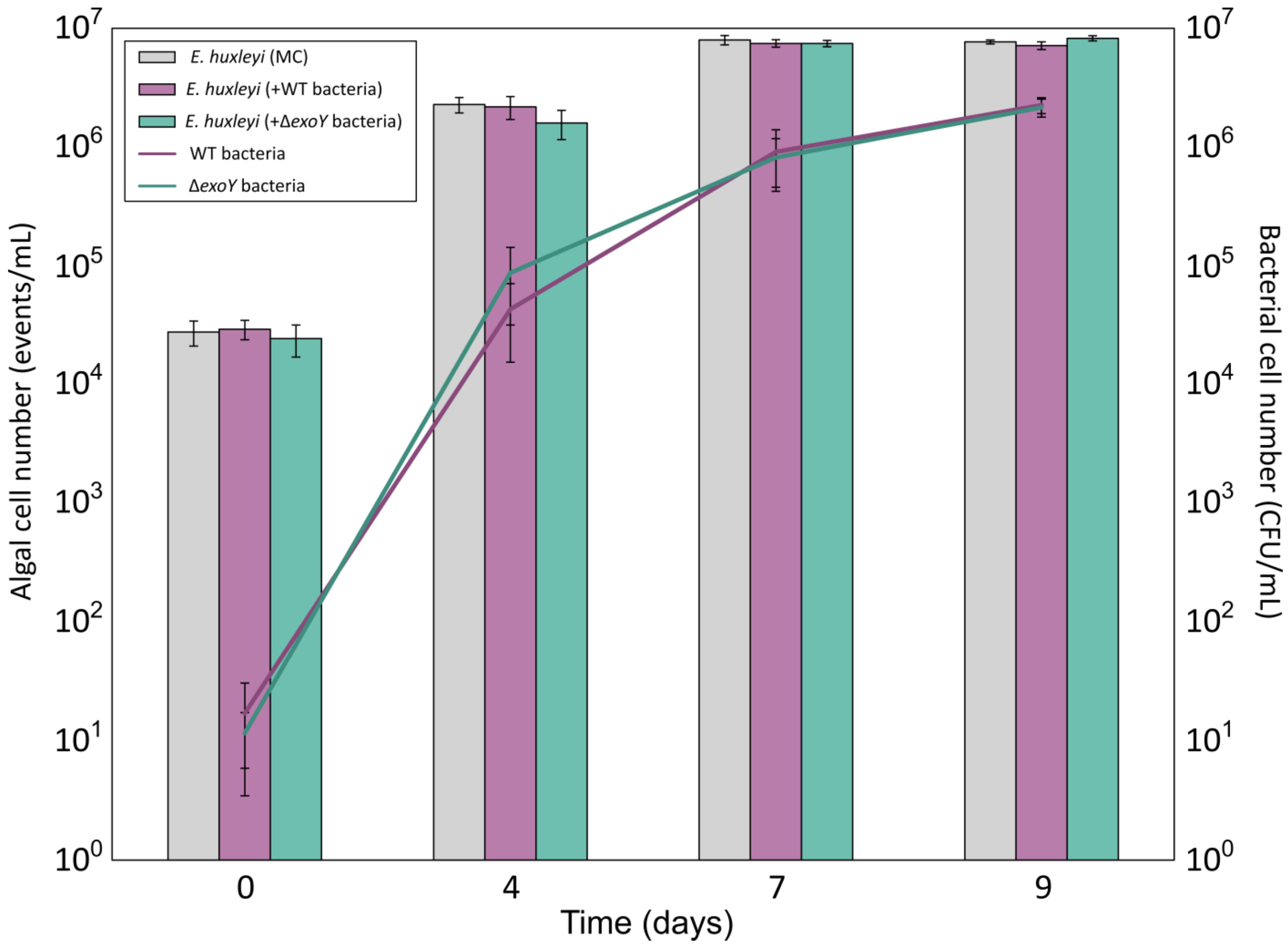

Supplementary Figure 10. Growth curves of algae and bacteria in co-cultures. *P. inhibens* WT or Δ*exoY* bacteria were grown with *E. huxley*i in liquid co-cultures. Algal growth was measured by flow cytometry (bar plots) and bacterial growth by plating and counting CFU/mL (lines). Error bars represent standard deviation for n = 3 biological replicates.

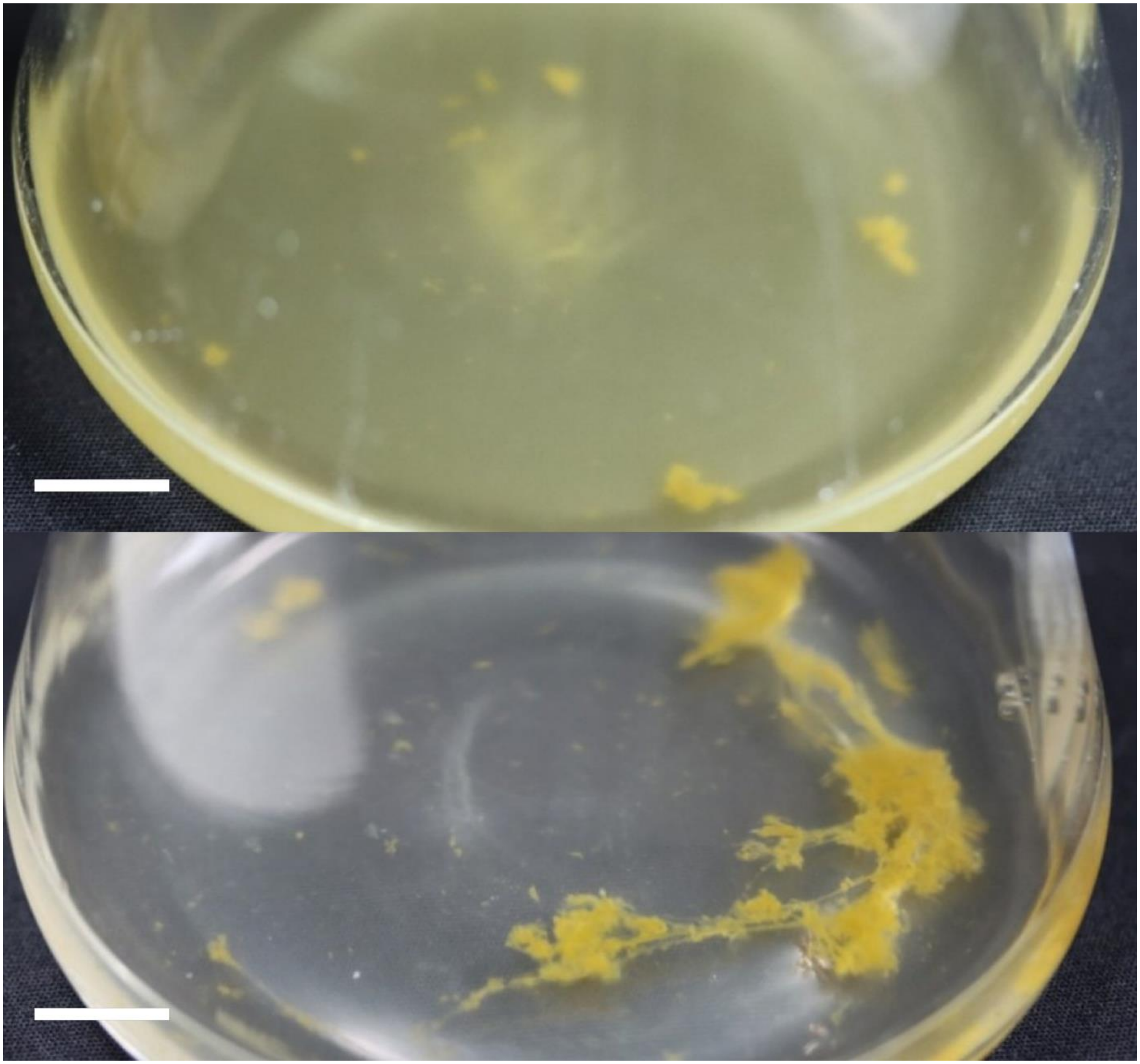

Supplementary Figure 11. *P. inhibens- E. huxleyi* co-cultures show higher aggregation compared to *E. huxleyi* monocultures. *E. huxleyi* monocultures grown to early stationary phase show dispersed cultures with few aggregates (top), while *P. inhibens - E. huxleyi* co-cultures of the same age show visibly higher aggregation. Scale bar represents 10 mm.

Supplementary Table 1. Genes related to key processes during the transition of *P. inhibens* bacteria from motile to sessile states. Genes potentially involved in motility, attachment and EPS production were selected based on homologous pathways in other characterized bacteria.

Group	Name	Gene ID	Function
Attachment	*dltA1*	PGA1_c13760	Biosynthesis of D-alanyl-lipoteichoic acid, involved in surface attachment in *S. aureus*[4,5].
EPS Production	*exoB*	PGA1_c34470	Biosynthesis of succinoglycan, involved in EPS production, in *S. meliloti*[6].
	exoY$_C$	PGA1_c05260	
	exoY$_P$	PGA1_262p00380	
Motility	*motA*	PGA1_c35680	Flagellar motor protein, chemotaxis proteins, control for motility[7].
	motB	PGA1_c25180	

Supplementary Table 2. Genetic modules of wzx/wzy-dependent EPS. Genes of characterized wzx/wzy-dependent EPS biosynthesis modules including colanic acid, xanthan, acetan, Pss, succinoglygan and amylovoran, in key producing bacteria and the corresponding genes in *P. inhibens* (found on the native 262kb plasmid and on the chromosome). Shown are the names and functional annotation of genes involved in addition of the priming sugar to the lipid carrier, repeat sugar chain biosynthesis, polymerization of the repeating units and transport. Functional annotations were generated using EggNOG-mapper v2[8], and the identified clusters of orthologous groups (COG) number is specified.

		**Repeat chain biosynthesis and assembly**						**Repeating units polymerization and secretion**							
		**Priming glycotransferases**			**Chain glycosyltransferases**			**Repeating unit flippase**		**Repeating unit polymerase**		**Polysaccharide copolymerase**		**Outer membrane polysaccharide export**	
**EPS**	**Producing Bacteria**	**Gene**	**EggNOG**	**Priming sugar**	**Gene**	**EggNOG**	**Chain sugars**	**Gene**	**EggNOG**	**Gene**	**EggNOG**	**Gene**	**EggNOG**	**Gene**	**EggNOG**
**Colanic acid**[9]	*Escherichia coli*<br>K-12 MG1655	*wcaJ*	COG2148	Glc	*wcaACEIL*	COG0297<br>COG0438<br>COG0463<br>COG1216	Fuc<br>Gal<br>Glc	*wzxC (wzx)*	COG2244	*wcaD (wzy)*	28KKV	*wzc*	COG0489<br>COG3206	*wza*	COG1596
**Xanthan**[9]	*Xanthomonas campestris* pv. *campestris* B100	*gumD*	COG2148	Glc	*gumHIKM*	COG0438<br>COG1922	Glu<br>Glc<br>Man	*gumJ*	COG2244	*gumE*	2BVF4	*gumC*	COG3206	*gumB*	COG1596
**Acetan**[10]	*Komagataeibacter xylinus*	*aceA*	COG2148	Glc	*aceBCKPQR*	COG0438<br>COG0463<br>COG1216<br>COG1922	Glu<br>Man<br>Rha	*aceE*	COG2244	*aceG*	2BVF4	*aceD*	COG0489<br>COG3206	*aceH*	COG1596
**Pss**[11]	*Rhizobium johnstonii*	*pssA*	COG2148	Glc	*pssCDEJS*	COG0438<br>COG0463<br>COG0707<br>COG1216<br>COG5017	Glc<br>Gal	*pssL*	COG2244	*pssT*	COG3307	*pssP*	COG0489<br>COG3206	*pssN*	COG1596
**Succinoglycan**[6]	*Sinorhizobium meliloti* 1021	*exoY*	COG2148	Gal	*exoALMOUW*	COG1215<br>COG0438<br>COG0463	Glc	*exoT*	COG2244	*exoQ*	COG3307	*exoP*	COG0489<br>COG3206	*exoF*	COG1596
**Amylovoran**[12]	*Erwinia amylovora*<br>CFBP1430	*amsG*	COG2148	Gal	*amsBDEK*	COG0438<br>COG1215<br>COG1216	Gal<br>Glc	*amsL*	COG2244	*amsC*	2DR4Q	*amsA*	COG0489<br>COG3206	*amsH*	COG1596
***P. inhibens* 262kb plasmid module**	*Phaeobacter inhibens* DSM 17395	PGA1_262p00380<br>*(exoY)*	COG2148		PGA1_262p00050<br>PGA1_262p00060<br>PGA1_262p00100<br>PGA1_262p00110<br>PGA1_262p00120<br>PGA1_262p00130<br>*(exoALMO)*	COG0438<br>COG0463<br>COG1215<br>COG1216		PGA1_262p00370	COG2244	PGA1_262p00070<br>*(exoQ)*	COG3307	PGA1_262p00090<br>*(exoP)*	COG0489<br>COG3206	PGA1_262p00580	COG1596
***P. inhibens* chromosomal module**	*Phaeobacter inhibens* DSM 17395	PGA1_c05260<br>(*exoY*-like)	COG2148		PGA1_c05300<br>PGA1_c05310<br>PGA1_c05360<br>PGA1_c05390<br>PGA1_c05400<br>PGA1_c05410	COG0438<br>COG0463<br>COG1215<br>COG1216<br>COG1922		PGA1_c05290<br>(*exoT*-like)	COG2244	PGA1_c05380	COG3307	PGA1_c05270<br>PGA1_c05280	COG0489<br>COG3206	PGA1_c08890	COG1596

Supplementary References

1. Sperfeld, M. *et al.* Bacterial lag phase shortening is triggered by methyl groups. *bioRxiv* (2023) doi:10.1101/2023.06.06.543872.

2. Love, M. I., Huber, W. & Anders, S. Moderated estimation of fold change and dispersion for RNA-seq data with DESeq2. *Genome Biol.* **15**, (2014).

3. Eliason, O. & Segev, E. Coccolith Sr/Ca is a robust temperature and growth rate indicator that withstands dynamic microbial interactions. *Geobiology* **20**, 435–443 (2022).

4. Thole, S. *et al. Phaeobacter gallaeciensis* genomes from globally opposite locations reveal high similarity of adaptation to surface life. *ISME J.* **6**, 2229–2244 (2012).

5. Gross, M., Cramton, S. E., Götz, F. & Peschel, A. Key role of teichoic acid net charge in *Staphylococcus aureus* colonization of artificial surfaces. *Infect. Immun.* **69**, 3423–3426 (2001).

6. Halder, U., Banerjee, A. & Bandopadhyay, R. Structural and Functional Properties, Biosynthesis, and Patenting Trends of Bacterial Succinoglycan: A Review. *Indian J. Microbiol.* **57**, 278–284 (2017).

7. Minamino, T., Imada, K. & Namba, K. Molecular motors of the bacterial flagella. *Curr. Opin. Struct. Biol.* **18**, 693–701 (2008).

8. Cantalapiedra, C. P., Herņandez-Plaza, A., Letunic, I., Bork, P. & Huerta-Cepas, J. EggNOG-mapper v2: Functional Annotation, Orthology Assignments, and Domain Prediction at the Metagenomic Scale. *Mol. Biol. Evol.* **38**, 5825–5829 (2021).

9. Vandana, G. & Das, S. Genetic regulation, biosynthesis and applications of extracellular polysaccharides of the biofilm matrix of bacteria. *Carbohydr. Polym.* **291**, 119536 (2022).

10. Trček, J., Dogsa, I., Accetto, T. & Stopar, D. Acetan and acetan-like polysaccharides: Genetics, biosynthesis, structure, and viscoelasticity. *Polymers (Basel).* **13**, 1–16 (2021).

11. Janczarek, M., Rachwał, K. & Kopcińska, J. Genetic characterization of the *Pss* region and the role of *PssS* in exopolysaccharide production and symbiosis of *Rhizobium leguminosarum bv. trifolii* with clover. *Plant Soil* **396**, 257–275 (2015).

12. Langlotz, C., Schollmeyer, M., Coplin, D. L., Nimtz, M. & Geider, K. Biosynthesis of the repeating units of the exopolysaccharides amylovoran from *Erwinia amylovora* and stewartan from *Pantoea stewartii*. *Physiol. Mol. Plant Pathol.* **75**, 163–169 (2011).
